# Hybrid Physics-Informed and Bayesian Modeling of Single-Nanoparticle–Cell Adhesion Kinetics under Cytoskeletal Perturbation

**DOI:** 10.34133/csbj.0077

**Published:** 2026-05-11

**Authors:** Houari Bettahar, Hélder A. Santos, Quan Zhou

**Affiliations:** ^1^ Department of Electrical Engineering and Automation, Aalto University, 02100 Espoo, Finland.; ^2^ Drug Research Program, Division of Pharmaceutical Chemistry and Technology, Faculty of Pharmacy, University of Helsinki, FI-00014 Helsinki, Finland.; ^3^ Department of Biomaterials and Biomedical Technology, The Personalized Medicine Research Institute (PRECISION), University Medical Center Groningen (UMCG), University of Groningen, 9713 AV Groningen, the Netherlands.

## Abstract

Understanding the dynamic mechanical interaction between nanoparticles and cell membranes is essential for advancing nanomedicine, yet modeling these kinetics is often hindered by biological variability, experimental noise, and the limitations of traditional equilibrium-based frameworks. In this study, we present an advanced hybrid Physics-Informed Neural Network (PINN) framework designed to capture the early-stage adhesion dynamics. Our architecture integrates an analytical Standard Linear Solid model as a mechanistic backbone to represent baseline viscoelasticity, augmented by a neural residual term that captures nonlinear, stochastic, and nonequilibrium deviations. To handle heterogeneous experimental data, we incorporate an adaptive, uncertainty-aware loss weighting scheme and a physics-informed Bayesian framework utilizing a heteroscedastic likelihood for robust parameter inference. We validated this approach using single-nanoparticle force measurements on fibroblasts and MiaPaCa-2 cancer cells under a range of pharmacological treatments (Chlorpromazine, Genistein, and Nocodazole). Validated via leave-one-out cross-validation, the hybrid model demonstrates condition-dependent predictive improvements over classical models, most pronounced in severely perturbed biological states where cytoskeletal disruption renders adhesion dynamics highly irregular. This approach offers a framework for cross-condition phenotyping, providing physically consistent parameter estimates across the full spectrum of cytoskeletal perturbation severity studied here. This work introduces an interpretable approach for modeling complex biophysical adhesion processes and offers a potentially generalizable framework for analyzing noisy, heterogeneous biological systems.

## Highlights

• A hybrid Physics-Informed Neural Network models nanoparticle–cell adhesion
• Neural correction enhances viscoelastic modeling beyond the SLS framework
• Adaptive uncertainty weighting balances data fidelity and physical consistency
• Bayesian inference quantifies uncertainty in adhesion forces and kinetic parameters
• Reveals treatment-specific adhesion signatures under cytoskeletal perturbation

## Graphical Abstract

**Figure:**
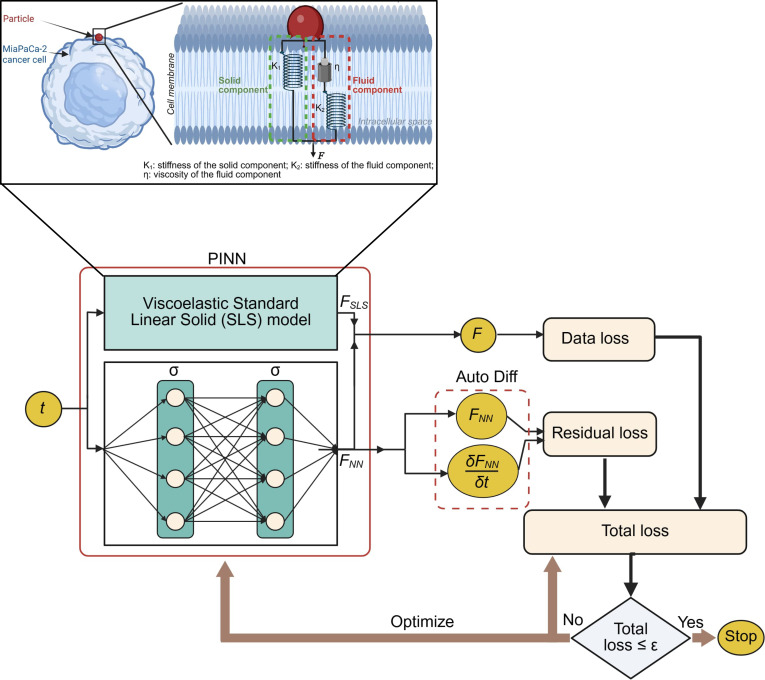

## Introduction

Understanding the kinetics of nanoparticle adhesion to cell membranes is critical for advancing nanomedicine, particularly in areas such as targeted drug delivery [1–3], cancer diagnostics [4,5], and mechanobiology [6]. This adhesion process governs how synthetic carriers interact with cellular surfaces, initiate uptake, and ultimately influence intracellular fate [7–9]. Mechanical forces arising at the membrane–particle interface not only reflect the molecular machinery driving adhesion but also encode information about cellular state [10], cytoskeletal activity [11], and membrane organization [12]. Therefore, capturing the dynamics of force development during adhesion can yield valuable insights into both fundamental cell biology and applied biomedical engineering.

Traditional modeling of nanoparticle–cell interactions typically relies on 4 primary frameworks: compartmental kinetic models, energy-based models, molecular dynamics (MD) simulations, and transport/dosimetry models [13]. While ordinary differential equation (ODE)-based compartmental models effectively track population-level flux [14–16], they neglect stochastic fluctuations, force dynamics, and mechanical dependencies, giving only a population-level view without capturing underlying biophysical mechanisms [17]. Energy-based models, predominantly derived from the Helfrich Hamiltonian, characterize the energetic competition between receptor-mediated adhesion and the elastic cost of membrane bending [18–23]. However, these models assume small local curvature and particles much larger than the membrane thickness. They treat membranes as purely elastic, neglecting viscoelasticity and cytoskeletal remodeling, and they typically capture static equilibrium states rather than dynamic processes [24,25]. To address some of these limitations, Gonzalez-Rodriguez and Barakat [26] proposed a theoretical framework incorporating receptor–ligand binding, membrane bending, cytoskeletal viscoelasticity, and force-dependent bond dynamics, drawing inspiration from the membrane detachment work of Dembo et al. [27]. Their model is based on force balance rather than energy minimization, but still assumes comparable receptor and ligand densities and omits receptor diffusion. It also remains primarily simulation-based and studied within the equilibrium states.

While atomistic and coarse-grained MD simulations offer high-resolution modeling of wrapping trajectories [28–30], they are computationally expensive, constrained to simplified particle geometries, and restricted to nanosecond–microsecond timescales, preventing them from modeling biologically relevant long-term and large-scale dynamics. Similarly, dosimetry models predict sedimentation and delivered dose [16] but lack the mechanical resolution to capture force-driven uptake. Consequently, a pervasive limitation across these approaches is their reliance on quasistatic equilibrium adhesion events. They overlook active, stochastic, and fundamentally nonequilibrium mechanical effects and cannot capture the time-dependent evolution of adhesion strength. A further constraint in advancing mechanistic understanding is the lack of quantitative experimental data on adhesion strength at the nanoparticle–cell membrane interface, which is essential for parameterizing and validating predictive models [31].

Recently, several data-driven approaches have emerged to bridge the gap between theory and observation. Traditional methods, such as parametric fitting [32], hierarchical Bayesian models [33,34], and ODE-constrained regression [35], are effective when the governing model structure is well-specified; however, they become mechanistically restrictive when key physical processes are oversimplified or only partially known. Conversely, while Physics-Informed Neural Networks (PINNs) have demonstrated efficacy in solving inverse problems [36,37] and handling data sparsity via uncertainty-aware loss weighting [38,39] or Bayesian inference [40,41]. These methods encounter a substantial representational bottleneck in biophysics. Standard PINN architectures typically treat the neural network as the primary solver, relying on it to approximate the entire solution space. Without a robust mechanistic backbone, they struggle to generalize in data-sparse biological regimes, often requiring excessive data to relearn fundamental rheological principles, such as viscoelasticity, from scratch.

To address these limitations, we introduce a hybrid PINN framework to capture the dynamics during the very early stage of nanoparticle–cell contact (0 to 12 min), using nanoparticles with diameters of 700 to 900 nm [42–44]. Rather than using a neural network to learn the full solution solely through a neural representation constrained by a physics-based loss [45,46], we explicitly embed an analytical Standard Linear Solid (SLS) model as a mechanistic backbone and augment it with a neural residual term. The SLS component provides a physically interpretable baseline for the early-time mechanical response of the membrane–cortex system, where viscoelastic deformation and membrane tension dominate [47–50] and are reasonably approximated by linear viscoelasticity. Nonlinear, stochastic, and nonequilibrium deviations from this baseline are captured by the neural residual component within the PINN framework. This hybrid architecture reduces the need for the network to relearn fundamental viscoelastic behavior from scratch and provides a mechanistically grounded prior for describing adhesion kinetics at the nanoparticle–cell interface. Furthermore, we incorporate uncertainty-aware loss weighting into the PINN framework, allowing it to balance fidelity to physical constraints with sensitivity to experimental noise and heterogeneity. To quantify confidence in both model predictions and learned parameters, we also develop a physics-informed Bayesian framework with a heteroscedastic likelihood, enabling robust posterior inference over time-evolving forces and biophysical parameters governing adhesion strength.

## Methods and Materials

### Experimental setup, experimental procedure, and datasets

**Fig. 1.**
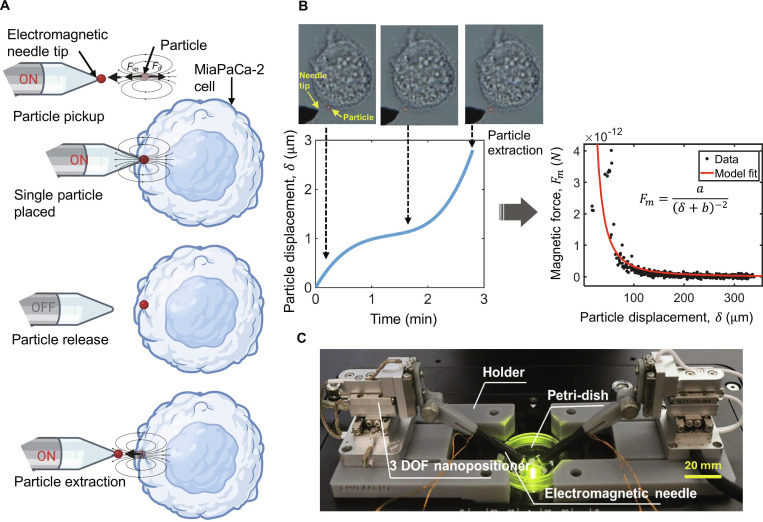
Single-nanoparticle manipulation and adhesion force measurement using an electromagnetic robotic needle system mounted on the microscope. (A) Schematic illustrating the process steps of particle manipulation: pickup of a single nanoparticle by the electromagnetic needle tip (ON), placement onto a cell membrane, release (OFF), and then re-engagement (ON) to extract the particle and measure the force. (B) Adhesion force measurement during particle extraction from the cell membrane. The extraction force $\left(F_{m}\right)$ is quantified by monitoring the particle’s displacement (δ) relative to the needle tip, using the calibrated magnetic field model $F_{m}=\frac{a}{{\left(\delta +b\right)}^{2}}$. (C) Experimental setup showing the dual-needle micromanipulation platform with 3-DOF nanopositioners, a Petri dish, and the electromagnetic needle positioned under an inverted microscope.

The experimental procedure consists of key steps (see Fig. 1A). First, a single magnetic submicroparticle (700 to 900 nm carboxyl-functionalized magnetic submicrometer particles) is captured by turning the electromagnetic needle “ON” and navigating it toward the particle (pickup) via high-precision XYZ nano-positioners. The captured particle is then transported and placed onto the membrane of a target cell while maintaining the magnetic field (placement). Once positioned, the magnetic field is deactivated (“OFF”) to allow passive interaction between the nanoparticle and the cell membrane (release). The particle is held on the membrane for a defined period, known as the adhesion time, after which it is extracted (see Fig. 1B).

The experimental setup is designed for the precise manipulation and force measurement of individual magnetic nanoparticles interacting with live adherent cells (see Fig. 1C). The system integrates robotic electromagnetic needles (0.5 mm diameter, 2 μm tip radius) mounted on 3-DOF (XYZ) nanopositioners (SLC1720, SmarACT) that enable subnanometer resolution movement in 3 dimensions. These needles generate localized magnetic fields when activated by a constant current (typically 0.75 A), allowing for dynamic control over nanoparticle capture, positioning, and release. A Petri dish containing cultured cells and dispersed magnetic nanoparticles is secured within a custom holder at the center of the workspace. The interaction site is illuminated with blue/green light-emitting diode light to enhance contrast for real-time microscopic monitoring [42,51].

To quantify these interactions during the extraction phase, a vision-based tracking system was used, which utilizes a high-speed camera and a vision algorithm based on a feature-matching technique to monitor nanoparticle dynamics. This system tracks the frame-by-frame movement of the particles, accounting for localized image features to maintain high data fidelity. These vision-derived spatial coordinates are subsequently used to compute the instantaneous distance δ from the magnetic needle, allowing for the estimation of the time-dependent force profile $F_{m}$ via a calibrated inverse square law: $F_{m}=\frac{a}{{\left(\delta +b\right)}^{2}}$, where aandb are empirically determined constants that characterize the magnetic field gradient and the particle’s magnetic moment. This empirical relationship is grounded in the dipole–field interaction model of the electromagnetic field gradient: $F_{m}=-\frac{4m_{p}\mu_{0}\beta M_{n}}{{\left(4\beta \delta +1\right)}^{2}}$. In this model, the fitting parameter a represents the combined magnetic and geometric properties $\left(m_{p},\mu_{0},\beta ,M_{n}\right)$, while b accounts for the effective magnetic offset $\left(\frac{1}{4\beta }\right).$ Here, $m_{p}$ is the particle’s magnetic moment, $\mu_{0}$ is the vacuum permeability, $M_{n}$ is the needle magnetization, and β is a geometric field gradient constant. By correlating these visual trajectories with the force model, both the particle’s displacement and the resulting extraction force are precisely measured [42,52].

The dataset generated from these experiments consists of adhesion force measurements collected across multiple time points and biological conditions [42]. Specifically, experiments were conducted on 2 cell types: MiaPaCa-2 pancreatic cancer cells and fibroblasts.

Fibroblast (*Homo sapiens*, foreskin) and MiaPaCa-2 (human pancreatic carcinoma epithelial) cells were cultured under well-defined conditions. Fibroblasts were maintained in DMEM (Dulbecco’s Modified Eagle Medium) supplemented with 10% heat-inactivated fetal bovine serum (FBS), 1% nonessential amino acids, 1% L-glutamine, 1% penicillin–streptomycin, and 1% sodium pyruvate, at 37 °C, 5% CO₂, and 95% humidity, with subculturing approximately once per week at confluency. MiaPaCa-2 cells were cultured in high-glucose DMEM supplemented with 10% heat-inactivated FBS, 2.5% horse serum, 1% penicillin–streptomycin, 4 mM L-glutamine, 1 mM sodium pyruvate, and 1,500 mg/l sodium bicarbonate, with subculturing at ~80% confluency. Cells were detached using standard trypsin-EDTA protocols, centrifuged, resuspended, and reseeded.

The experiments used standardized 700- to 900-nm carboxyl-functionalized magnetic submicrometer particles (1% w/v), which provide a high signal-to-noise ratio for force estimation without the confounding variables of complex surface modifications.

The experimental conditions included the following: (a) control MiaPaCa-2 cells (no inhibitor), (b) control fibroblasts (no inhibitor), (c) chlorpromazine-treated MiaPaCa-2 cells (10 μM, 30-min incubation in a 5% CO_2_ incubator, 37 °C, 95% humidity) to inhibit clathrin-mediated endocytosis, (d) genistein-treated MiaPaCa-2 cells (100 μM, 30-min incubation in a 5% CO_2_ incubator, 37 °C, 95% humidity) to inhibit caveolae-mediated endocytosis, (e) nocodazole-treated MiaPaCa-2 cells (10 μM, 30-min incubation in a 5% CO_2_ incubator, 37 °C, 95% humidity) to disrupt microtubule dynamics, and (f) combined-inhibitor-treated MiaPaCa-2 cells (30-min incubation in a 5% CO_2_ incubator, 37 °C, 95% humidity), where multiple pathways were simultaneously disrupted to assess cumulative effects on nanoparticle adhesion and uptake. All inhibitor treatments were prepared from stock solutions and included vehicle controls (e.g., 0.1% DMSO for Nocodazole/Genistein) to ensure observed differences reflected intended pathway perturbations rather than nonspecific effects.

For each condition and each adhesion time (2, 4, 6, 8, and 10 min), 4 to 5 independent measurements were recorded using different nanoparticles and different cells. All measurements were performed on different nanoparticles and across various cell types, introducing important biological and material variability. After each interaction, the extraction force, corresponding to the detachment strength, was recorded by re-engaging the magnetic needle. This protocol enables the precise probing of time-dependent adhesion dynamics and the role of intracellular trafficking pathways in nanoparticle–membrane interactions.

This heterogeneity, arising from the use of different cell types and nanoparticle properties, poses considerable challenges for conventional modeling techniques, which often struggle to capture the complex, nonlinear, and uncertain dynamics of such systems. To address these difficulties, the dataset is reused here to implement a hybrid modeling approach combining classical viscoelastic models with PINNs to capture both the mechanical and biological dynamics more accurately. Furthermore, the framework incorporates an uncertainty-aware loss weighting strategy and a physics-informed Bayesian model, enabling reliable posterior inference of adhesion forces and biophysical parameters even under heterogeneous and noisy experimental conditions.

### Modeling

#### PINN formulation for adhesion kinetics

**Fig. 2.**
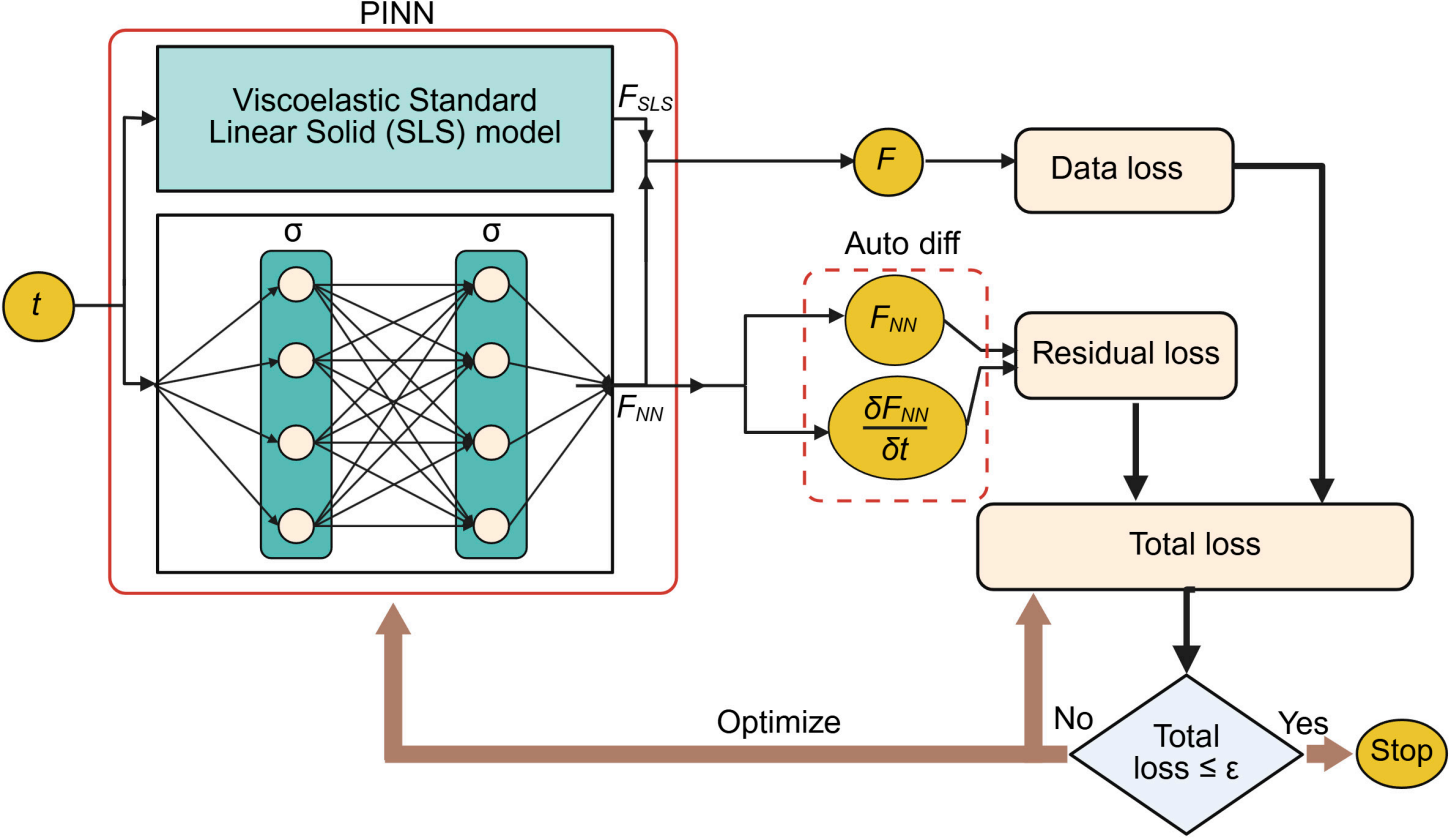
Schematic of the Physics-Informed Neural Network (PINN) framework integrating the SLS model for viscoelastic adhesion force modeling. The network takes time t as input and outputs the predicted force F, composed of an analytical SLS component and a neural correction term. The total loss combines a data loss, based on the discrepancy between predicted and measured forces, and a physics (residual) loss, which penalizes violations of the governing differential equation derived from the SLS model. The network parameters are optimized until the total loss falls below a predefined threshold ε, ensuring that the learned model is both data-consistent and physically grounded.

The membrane experiences several types of stresses during nanoparticle internalization such as cytoplasmic viscoelastic stress, elastic stress from membrane–particle bonds, and short-range repulsion [26,53]. While these components can be described analytically, traditional linear frameworks often rely on quasi-equilibrium assumptions and cannot capture the inherently nonequilibrium and stochastic nature of cellular adhesion. To address these limitations, the hybrid PINN framework combines a physically interpretable SLS backbone (see Note S1 and Fig. S1), which captures the dominant early-time viscoelastic response, with a neural residual term that learns deviations arising from stochastic, nonlinear, and active cellular processes, as schematically shown in Fig. 2. In this framework, the physical behavior of the system is described by the analytical SLS model, which governs the time evolution of the viscoelastic force, $F_{\mathrm{SLS}}\left(t\right)$. The total force prediction is defined as the sum of the SLS component and the neural network correction:

F^tiθ=FSLStiθphys+FNNtiθnn (1)

where $\theta_{\text{phys}}$ represents the parameters of the physical model ($F_{\max},F_{0},a$), and $F_{\mathrm{NN}}$ is a neural network trained to capture data-driven deviations. $\theta_{\mathrm{nn}}\in R^{p}$, where p is the total number of trainable weights and biases in the neural network layers. The total loss function consists of a data term and a physics-informed term:

Lθ=Ldata+Lphys (2)

with the data loss given by the Smooth-L1 (Huber) function (defined in Note S3):

Ldata=1N∑i=1NSmooth−L1FdatatiF^tiθ (3)

The physics-informed term $L_{\text{phys}}$ is implemented as a regularization on the neural network component $F_{\mathrm{NN}}$. Because the SLS component $F_{\mathrm{SLS}}$ satisfies the governing ODE by construction, $L_{\text{phys}}$ serves to minimize the energy and complexity of the neural correction. To ensure numerical commensurability with the data loss and to prevent the physics term from dominating training due to scale differences in force units, $L_{\text{phys}}$ is normalized by the empirical variance of the observed force data:

Lphys=1VarFdata·1M∑j=1MF^NNtj2+dF^NNtjdt2 (4)

where M is the number of collocation points. The first term penalizes the magnitude of the neural correction, encouraging it to remain small unless the data genuinely requires a departure from the SLS backbone. The second term penalizes the temporal rate of change of the correction, discouraging rapid oscillations that would be physically implausible. The variance normalization $\mathrm{Var}\left(F_{\text{data}}\right)$ is computed once from the training data before optimization begins and held fixed throughout training, placing both $L_{\text{data}}$ and $L_{\text{phys}}\;$ on a commensurate numerical scale from epoch 1.

The derivative $\frac{d{\hat{F}}_{\mathrm{NN}}}{\mathrm{dt}}$ is computed using automatic differentiation (Autograd) via the PyTorch engine. This allows for exact gradient computation of the neural network layers with respect to the input time t during the training process, rather than relying on numerical approximations.

Although the SLS component is linear in time, the inclusion of a neural network residual introduces nonlinear expressivity to the model. The neural correction term $F_{\mathrm{NN}}$ is implemented as a fully connected multilayer perceptron (MLP) that takes normalized time as a one-dimensional input and outputs a scalar correction term. The network consists of 2 hidden layers with 32 neurons each and SiLU (Sigmoid Linear Unit) activation functions, followed by a linear output layer. Details of the neural network architecture, physics regularization, and training configuration of the PINN model are described in Note S5.

Incorporating a physics-informed loss term derived from the governing differential equation of the SLS model constrains the solution space to physically consistent trajectories. This regularization is particularly beneficial when the dataset is small or affected by noise, as is often the case with biological measurements, since it reduces overfitting and guides the model toward plausible solutions even in poorly sampled regions. By enforcing physical consistency, the physics loss improves generalization to unseen time points or experimental conditions, enhances robustness, and enables meaningful interpretation of model parameters. Therefore, while data loss ensures fidelity to the observed values, the addition of physics loss yields a model that is both accurate and physically grounded, even under challenging data conditions. In contrast, minimizing data loss alone allows the model to fit the measured adhesion force values, but this purely data-driven approach lacks constraints to ensure compliance with known physical laws. As a result, the model may overfit noisy or sparse data, leading to solutions that deviate from expected viscoelastic behavior.

#### Adaptive loss weighting with learned uncertainties

To balance the contributions of the data and physics-based losses during training, we employed an adaptive weighting scheme that learns the relative importance of each term based on their associated uncertainties. This approach is particularly useful when working with heterogeneous datasets (e.g., different nanoparticles and cell types), where the reliability of data and physical constraints can vary considerably across samples.

We define the total loss as a weighted sum of the data mismatch loss $L_{\text{data}}$ and the physics-informed residual loss $L_{\text{phys}}$, with the weights derived from learnable log-variances:

Lθ=1σd2Ldata+1σp2Lphys+logσd2+logσp2 (5)

where $\sigma_{d}^{2}$ and $\sigma_{p}^{2}$ are the trainable variances corresponding to the data and physics losses, respectively. Optimizing the loss in this form is equivalent to maximizing a Gaussian likelihood for each task under heteroscedastic uncertainty, as proposed by Cipolla et al. [54].

This formulation ensures that the variances $\sigma_{d}^{2}$ and $\sigma_{p}^{2}$ are always positive (since e(·)>0); it allows the model to automatically down-weight loss terms associated with higher uncertainty during training and provides a numerically stable and interpretable optimization process that avoids manual tuning of loss weights.

**Fig. 3.**
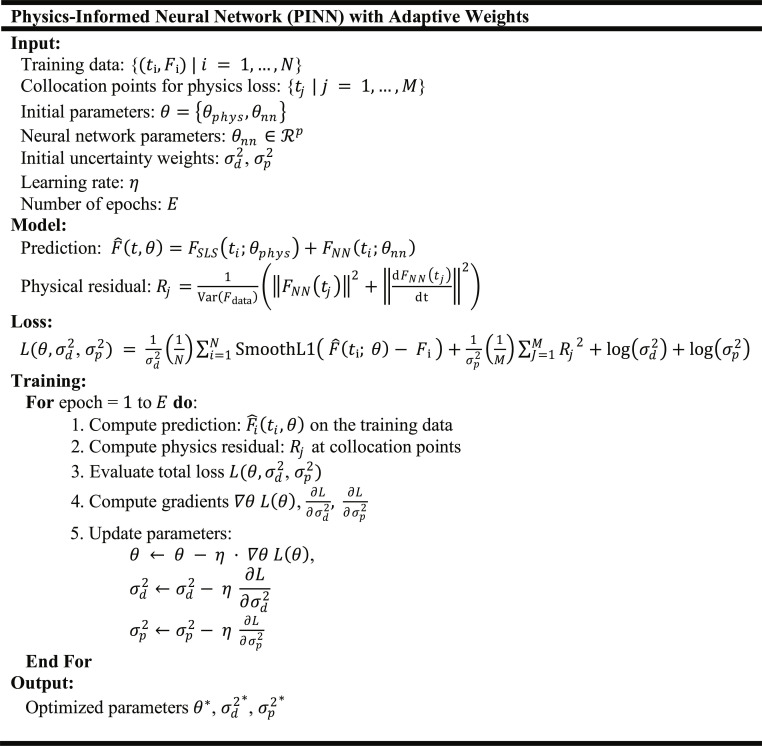
Algorithm of PINN with adaptive weights for modeling nanoparticle–cell adhesion dynamics. It integrates data loss with physics-based residuals from the SLS model, enabling uncertainty-aware training and parameter estimation.

The algorithm for the PINN with adaptive weights, which models nanoparticle–cell adhesion kinetics, is shown in Fig. 3. The input includes measured force–time data, collocation points for physics-based residuals, initial network parameters, uncertainty weights, learning rate, and the number of training epochs. The model predicts forces as the sum of an SLS model component and a neural network correction, while computing a physical residual to enforce consistency with underlying dynamics. The loss function combines a data-fitting term, computed using the Smooth-L1 function to reduce sensitivity to outliers (mathematically described in Note S3), a physics-based term weighted by model uncertainty, and logarithmic regularization of the uncertainties. During training, predictions, residuals, and total loss are evaluated iteratively, gradients are computed, and both neural network parameters and uncertainty weights are updated. The output consists of the optimized network parameters and uncertainties, yielding a calibrated, physics-informed model of nanoparticle–cell adhesion.

#### Physics-informed Bayesian modeling for adhesion kinetics

To quantify uncertainty in the parameters governing the force evolution during nanoparticle–cell adhesion, we adopt a Bayesian framework with a heteroscedastic likelihood model. While the Hybrid PINN (“PINN formulation for adhesion kinetics” section) serves as a discovery tool to reconstruct total force kinetics, including nonequilibrium neural residuals, this Bayesian stage is specifically designed to perform robust inference on the interpretable biophysical parameters of the mechanistic backbone. This probabilistic approach integrates prior knowledge, obtained from the SLS model, with experimental force data, enabling robust inference under noise and parameter uncertainty. The core of this methodology is based on Bayes’ theorem:

pθFdata=pFdataθ·pθpFdata (6)

where $\theta =\left\{F_{\max},\;F_{0},\;a\right\}$ represents the set of model parameters; $p\left(F_{\text{data}}|\theta \right)$ is the likelihood; $p\left(\theta \right)$ is the prior; $p\left(F_{\text{data}}\right)$ is the marginal likelihood (evidence); $p\left(\theta |F_{\text{data}}\right)$ is the posterior.

1. **Likelihood model**

The observed force data are modeled with a heteroscedastic normal likelihood:

$$F\left(t\right)∼N\left(\mu \left(t\right),\;\sigma \;\left(t\right)\right)$$ (7)

where N denotes the normal distribution (mathematically described in Note S2), and

μt=F0+Fmax−F01−e−tτ (8)

σt=γμμt+γσσlt (9)

$\mu \left(t\right)\;$ is the predicted mean force. $\sigma_{l}\left(t\right)$ is the empirical standard deviation and is estimated in sliding time windows. $\gamma_{\mu }$, $\gamma_{\sigma }$: Heteroscedasticity parameters: $\gamma_{\mu }$ controls signal–proportional noise (e.g., larger forces may have higher variability). $\gamma_{\sigma }$ scales baseline noise (e.g., experimental or biological variability).

2. **Priors distributions**

Priors for the model parameters follow an empirical Bayes approach, in which the prior centers for $F_{\max}$, $F_{0}$, and τ are informed by point estimates obtained from the deterministic SLS fitting step applied to the same dataset, while the remaining parameters use fixed priors independent of the fitting step. The prior distributions are specified as:

Fmax∼NFmax∗σFmax∗ (10)

F0∼N≥0F0∗σF0∗ (11)

τ∼HNa∗στ∗ (12)

γμ,γσ∼HCβ (13)

where $F_{\max}^{*},F_{0}^{*}$, and $\tau^{*}$denote the parameter estimates returned by the SLS fitting step for each condition. While this may introduce a degree of circularity, the prior serves only to initialize the search region; the posterior is subsequently shaped by the full probabilistic likelihood and Markov Chain Monte Carlo (MCMC) sampling across deliberately broad prior variances ($\sigma_{F_{\max}^{*}}$, $\sigma_{F_{0}^{*}}$ = 300 pN, and $\sigma_{\tau^{*}}$ = 1 min) relative to physically plausible ranges. Consequently, the likelihood dominates the posterior in data-rich regions, and the empirical Bayes centering functions mainly as a sampler initialization rather than a strong constraint. The prior for τ is set as Half Normal (HN) ($\sigma_{\tau^{*}}$ = 1 min), which is broad enough to cover all physically plausible adhesion time constants while enforcing positivity. The lower bounds (lower = 0 for $F_{0}$) enforce physical plausibility. Ndenotes a truncated normal distribution constrained to nonnegative values. $N_{\geq 0}$ denotes a truncated normal distribution constrained to nonnegative values. $\gamma_{\mu }$ and $\gamma_{\sigma }$ denote the heteroscedastic noise scale parameters, regularized via weakly informative Half-Cauchy priors with β=3, independent of the SLS fitting step; both are described mathematically in Note S2.

3. **Posterior sampling**

The posterior distribution $p\left(F_{\max},F_{0},a|F_{\text{data}}\right)$ is not analytically tractable. We use MCMC [55,56], specifically the No-U-Turn Sampler (NUTS) [57], to approximate it.

Instead of evaluating the posterior directly, we sample using its log-form**.** This formulation is numerically stable and gradient-friendly, which is critical for MCMC sampling:

logpθFdata=logpFdataθ+logpθ+constant (14)

where $\log\;p\left(F_{\text{data}}|\theta \right)$ is the sum of log-likelihoods across observations, and $\log\;p\left(\theta \right)$ is the sum of log-priors for all parameters.

NUTS samples from the posterior $p\left(\theta |F_{\text{data}}\right)$ without needing to compute the constant $p\left(F_{\text{data}}\right)$. It generates new candidate values of θ and accepts or rejects them based on the log-posterior, thereby balancing data fit and prior conformity. Posterior sampling was performed using 4 independent chains with 500 warmup steps and 500 draws per chain, yielding 2,000 total posterior samples per condition, with target acceptance probability set to 0.9 and initialization via an adaptive diagonal pre-conditioning scheme. Convergence was assessed using the rank-normalized Gelman–Rubin statistic ($\hat{R}$) and the bulk effective sample size (${\mathrm{ESS}}_{\text{bulk}}$) [58]. $\hat{R}$ ≤ 1.01 was achieved for all 18 parameter-condition combinations ($F_{0}$, $F_{\max}$, and *τ* across all 6 conditions), confirming adequate convergence of all 4 chains to a common posterior distribution. The minimum ${\mathrm{ESS}}_{\text{bulk}}$ was 751 ($F_{0}$, Chlorpromazine condition), with all remaining values exceeding 700, well above the recommended threshold of 400, confirming reliable posterior exploration throughout. Full convergence diagnostics are provided in Fig. S14, and per-chain trace plots confirming chain mixing for all conditions are provided in Figs. S8 to S13.

4. **Predictive distribution**

For unseen time points $t^{*}$, the predictive distribution integrates over parameter uncertainty:

pFt∗Fdata=∫pFt∗θpθFdatadθ (15)

This integral is approximated by averaging over N posterior samples ${\left\{\theta^{i}\right\}}_{i=1}^{N}$:

pFt∗Fdata≈1N∑i=0NpFt∗θi (16)

where each $\theta^{i}$ is drawn via MCMC from the log-posterior as described above. To ensure physical plausibility, negative force predictions are truncated, resulting in a truncated normal predictive distribution:

Ft∗∼TNμt∗σt∗ (17)

where TN denotes truncated normal (mathematically described in Note S2).

**Fig. 4.**
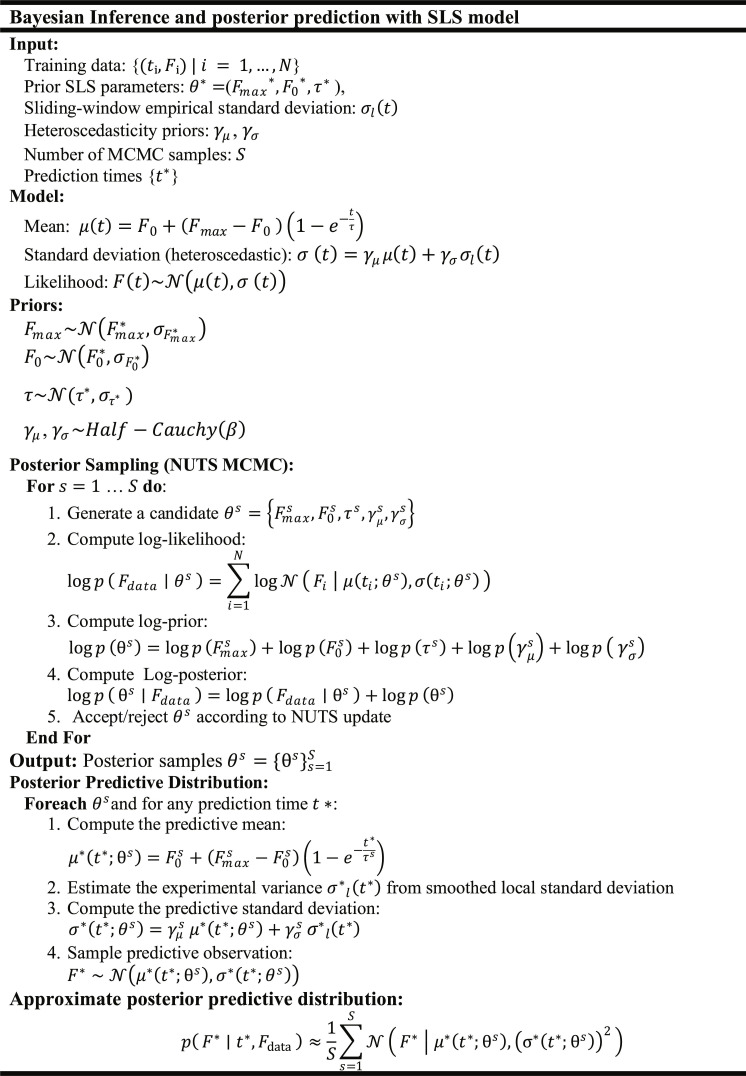
Algorithm of Bayesian inference and posterior prediction of single-nanoparticle–cell adhesion forces using an SLS model as prior. It incorporates heteroscedasticity, No-U-Turn Sampler (NUTS) sampling for posterior estimation, and sliding-window-based variance for predictive uncertainty.

Figure 4 illustrates the Bayesian inference algorithm with posterior prediction, using the SLS model as a prior. The input consists of the measured force–time data, prior estimates of SLS parameters, sliding-window empirical standard deviations, heteroscedasticity priors, the number of MCMC samples, and prediction times. The model assumes that the mean force follows the SLS equation and the heteroscedastic standard deviation accounts for both proportional and empirical variability. Priors for the SLS parameters are Gaussian, while heteroscedasticity parameters follow Half-Cauchy distributions (mathematically described in Note S2). Posterior sampling is performed using the NUTS MCMC algorithm, where candidate parameter sets are generated, and log-likelihood, log-prior, and log-posterior values are computed for acceptance or rejection. The output consists of posterior samples of all parameters. Using these samples, the algorithm computes the posterior predictive distribution for any desired prediction time, including predictive mean, predictive standard deviation, and sampled predictive observations, providing a fully probabilistic estimate of nanoparticle–cell adhesion dynamics.

## Results

### Impact of the neural network component and adaptive uncertainty-based loss weighting in PINNs on adhesion kinetics modeling

To evaluate the contribution of the neural network component and adaptive uncertainty-based loss weighting in PINNs, we examined and compared 3 modeling strategies for predicting adhesion force as a function of adhesion time across 6 experimental conditions: untreated fibroblast cells, untreated pancreatic cancer cells (MiaPaCa-2), and MiaPaCa-2 cells treated with specific endocytic or cytoskeletal inhibitors: Chlorpromazine, Genistein, Nocodazole, and a combination of all 3 inhibitors. The 3 evaluated strategies included the following: (a) a parameter-fitted SLS model (see pseudocode of the training algorithm in Fig. S2), (b) a nonadaptive SLS_PINN model with equally fixed weighting between data and physics loss components, and (c) an adaptive SLS_PINN model employing uncertainty-guided dynamic loss weighting (see pseudocode of the training algorithm in Fig. 3). The SLS_PINN models follow a hybrid structure, as defined in Eq. (10), in which the predicted force is modeled as the sum of an analytical SLS solution and a neural network correction term. Because the models are trained on experimentally measured force–time data, the inferred parameters are interpreted as effective descriptors of adhesion dynamics rather than direct measurements of specific molecular processes such as receptor recruitment, cytoskeletal remodeling, or signaling activity. Given the relatively small sample size of the dataset of 4 to 5 independent replicates per time point across 5 time points (*N* = 20 to 25 observations per condition), all biological interpretations of inferred parameter differences are regarded as mechanistically consistent, hypothesis-generating findings rather than statistically confirmed causal conclusions. The observed differences in kinetic parameters across conditions are supported by convergent evidence from the pharmacological literature and our previously published experimental data [42]. It should also be noted that direct cell viability assays and target-engagement verification, such as receptor occupancy measurements or downstream signaling readouts, were not performed as part of the present experimental protocol. The absence of such orthogonal measurements represents a residual limitation of this study; the model-inferred parameter differences across conditions should therefore be interpreted as effective kinetic phenotypes consistent with known pharmacological actions, rather than confirmed readouts of specific molecular engagement events.

The hybrid PINN was designed specifically to address the small-*N* constraint inherent to single-cell magnetic force measurements: the physics backbone provides structural regularization that constrains the solution space, reducing the degrees of freedom that the neural network must learn; the neural network correction term captures residual deviation from the physically constrained solution; and the Bayesian layer propagates measurement uncertainty directly into posterior parameter distributions. Together, these choices ensure robust parameter estimation and physically consistent predictions even when only 4 to 5 replicates per time point are available, a regime where purely data-driven approaches would be unreliable.

Model performance was assessed using 3 complementary metrics: the coefficient of determination (Loo-*R*^2^ ± SD, where SD was estimated via leave-one-out resampling), leave-one-out resampling log-likelihood (LOO-LL), and leave-one-out root mean square error (LOO-RMSE). Full details of how there are estimated can be found in Note S4, and the complete results are summarized in Table 1. Importantly, all 3 metrics are computed on the full set of raw replicate measurements at each time point, not on time-point means. This ensures that replicate-level variability is fully captured in every performance estimate. Loo-*R*^2^ differences are interpreted as meaningful only when they exceed the larger of the two compared SDs; cases where the LOO-*R*^2^ gap falls within the SD range are treated as statistically equivalent. LOO-LL and LOO-RMSE serve as independent confirmatory metrics. Crucially, LOO-RMSE is treated as the primary criterion for distinguishing genuine predictive improvement from prior-induced bias, since it directly measures out-of-sample prediction error without refitting and is therefore sensitive to overregularization by an inadequate physics prior.

**Table 1.** Comparison of model fitting performance across 6 cellular adhesion conditions: Loo-*R*^2^ ± SD was estimated using leave-one-out resampling. Log-likelihood was computed assuming normally distributed residuals, also via leave-one-out resampling (Loo-LL). LOO-RMSE (pN) quantifies out-of-sample predictive error. Higher *R*^2^ and log-likelihood values and lower LOO-RMSE indicate better model performance. Differences in LOO-*R*^2^ smaller than the larger of the two compared SDs should not be interpreted as meaningful.

Dataset	Model	LOO-*R*^2^ *±* SD	LOO-LL	LOO-RMSE
Fibroblast (no treatment)	SLS-PINN w/ adapt	0.742 ± 0.198	−78.57	45.57
	SLS-PINN w/o adapt	0.742 ± 0.207	−78.57	45.56
	SLS	0.812 ± 0.142	−76.18	38.85
Mia-PaCa-2 (no treatment)	SLS-PINN w/ adapt	0.552 ± 0.101	−130.37	120.19
	SLS-PINN w/o adapt	0.452 ± 0.144	−132.48	132.91
	SLS	0.411 ± 0.106	−133.25	137.84
Chlorpromazine treated	SLS-PINN w/ adapt	0.449 ± 0.202	−132.11	178.83
	SLS-PINN w/o adapt	0.501 ± 0.211	−131.12	170.25
	SLS	0.160 ± 0.110	−136.33	220.82
Genistein treated	SLS-PINN w/ adapt	0.639 ± 0.130	−165.15	138.81
	SLS-PINN w/o adapt	0.633 ± 0.136	−165.39	140.11
	SLS	0.601 ± 0.073	−166.45	145.92
Nocodazole treated	SLS-PINN w/ adapt	0.371 ± 0.280	−174.67	67.73
	SLS-PINN w/o adapt	0.396 ± 0.285	−174.05	66.40
	SLS	0.055 ± 0.117	−180.99	83.05
Combination treated	SLS-PINN w/ adapt	0.272 ± 0.299	−97.39	54.15
	SLS-PINN w/o adapt	0.257 ± 0.318	−97.58	54.70
	SLS	0.030 ± 0.273	−99.97	62.49

As reported in our previous study [42], fibroblasts exhibited a progressive increase in adhesion forces under various experimental conditions. Specifically, clathrin inhibition increased adhesion by 39% and caveolae inhibition by 27%, while microtubule disruption reduced adhesion by 48%. Moreover, the combined inhibition of clathrin, caveolae, and microtubules led to a 70% reduction in adhesion. In the untreated case, MiaPaCa-2 cells and fibroblasts both exhibit the classic biphasic adhesion kinetics described as was also observed in other studies [59–62], suggesting a common mechanism across different cell types, but they differ markedly in nonlinearity and variance”.

#### Untreated MiaPaCa-2 cells

**Fig. 5.**
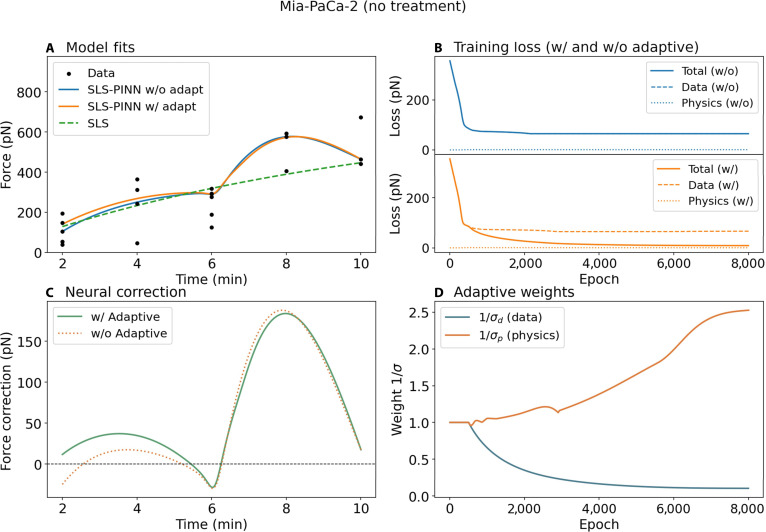
Model fitting and training dynamics for MiaPaCa-2 cells (no treatment). (A) Model fits to traction force measurements over time. Black dots represent experimental data points. The Standard Linear Solid Physics-Informed Neural Network without adaptive weighting (SLS-PINN w/o adapt, blue line) and with adaptive weighting (SLS-PINN w/ adapt, orange line) are compared against the deterministic SLS baseline (green dashed line). (B) Training loss curves for the nonadaptive (top) and adaptive (bottom) PINN variants, decomposed into total loss (solid line), data loss (dashed line), and physics regularization loss (dotted line). (C) Neural correction term, defined as the difference between the PINN prediction and the SLS backbone, for both adaptive and nonadaptive variants. Positive values indicate upward correction relative to the SLS fit. (D) Evolution of the adaptive loss weights $1/\sigma_{d}$ (data weight) and $1/\sigma_{p}$ (physics weight) over training epochs, reflecting the learned balance between data fidelity and physics regularization.

Figure 5A presents the model fits to force–time data obtained from untreated MiaPaCa-2 cells. MiaPaCa-2 cells show a steep, nonlinear force trajectory that rises from approximately 107 pN at 2 min to a peak of ~580 pN at 8 min, followed by a partial decrease toward ~450 pN at 10 min. The SLS model (LOO-*R*^2^ ± SD = 0.411 ± 0.106) fails to capture this nonmonotonic behavior entirely: it predicts a continuous, near-linear force increase across the full time window, reaching only ~450 pN at *t* = 10 min without any peak or reversal. This is a fundamental structural limitation of the SLS parametric form, which is constrained to monotonically increasing saturation curves and therefore cannot represent any force decrease in the late adhesion phase. The nonadaptive SLS_PINN yields LOO-*R*^2^ = 0.452 ± 0.144, a nominal gain of ΔLOO-*R*^2^= +0.041 over SLS. Critically, both PINN variants correctly capture the nonmonotonic force trajectory, rising sharply to ~580 pN at *t* = 8 min and then declining, closely tracking the observed data peak in a way the SLS model is structurally incapable of reproducing. Despite this qualitative improvement in structural representation, the quantitative metrics do not support a statistically meaningful performance advantage: the ΔLOO-*R*^2^= +0.041 is smaller than the SD of the nonadaptive PINN itself (±0.144), placing the 2 models within each other’s variability range. LOO-LL improves only marginally from −133.25 to −132.48 (ΔLOO-LL = +0.77), and LOO-RMSE remains elevated at 132.91 pN versus 137.84 pN for SLS (Δ = −5.07 pN). All 4 metrics are therefore consistent in indicating that the nonadaptive SLS_PINN offers no statistically meaningful, generalizable improvement over the SLS model for this condition, despite its superior ability to represent the qualitative shape of the force trajectory. Examination of the nonadaptive model’s training dynamics (Fig. 5B) reveals an early rapid drop in total loss followed by a prolonged plateau dominated by the data fidelity term, with the physics-based loss remaining near zero throughout training, a signature of rigid loss weighting preventing the model from dynamically balancing data fitting against physical plausibility.

The adaptive SLS_PINN yields LOO-*R*^2^ = 0.552 ± 0.101, a nominal gain of ΔLOO-*R*^2^ = +0.141 over SLS. Although the large SD (±0.101) means the confidence intervals substantially overlap and LOO-*R*^2^ alone cannot establish a reliable performance advantage, the directional consistency across the remaining 3 metrics provides stronger collective support: LOO-LL improves by ΔLOO-LL = +2.88 (from −133.25 to −130.37), and LOO-RMSE improves to 120.19 pN versus 137.84 pN for SLS (Δ = −17.65 pN). The convergent improvement across LOO-LL and LOO-RMSE, both of which penalize overfitting through out-of-sample evaluation, provides tentative multimetric support for the adaptive model capturing additional structure beyond the SLS backbone that generalizes beyond the training observations. The adaptive model is further distinguished by its training dynamics (Fig. 5B, lower panel; Fig. 5C): the physics weight $1/\sigma_{p}^{2}$ increases progressively from ~1.0 to ~2.3 across 8,000 epochs while the data weight $1/\sigma_{d}^{2}$ decreases from ~1.0 to ~0.1, confirming that the model correctly identifies the high data uncertainty in this condition and progressively redirects learning emphasis toward physical consistency. The total adaptive loss converges to a lower final value than the nonadaptive variant, indicating superior training stability. The neural correction term (Fig. 5D) peaks at approximately 175 pN near *t* = 8 min and then sharply decreases, with the adaptive correction consistently larger than the nonadaptive counterpart across the mid-to-late adhesion phase, consistent with the adaptive model applying a stronger, physically regularized correction precisely where force nonmonotonicity and data scatter are highest. Taken together, the 4-metric evaluation identifies the adaptive SLS_PINN as the preferred model for this condition, supported by LOO-LL and LOO-RMSE improvements, superior training stability, and a physically regularized correction that better captures the nonmonotonic force dynamics, even though LOO-*R*^2^ alone does not unambiguously establish this conclusion given the overlapping confidence intervals. Biologically, the nonmonotonic force trajectory of MiaPaCa-2 cells likely reflects cancer-cell-specific cytoskeletal organization and membrane dynamics that introduce force-buildup kinetics including a late-phase force relaxation, exceeding the structural capacity of the SLS framework under noisy, limited-density conditions.

#### Untreated fibroblast cells

**Fig. 6.**
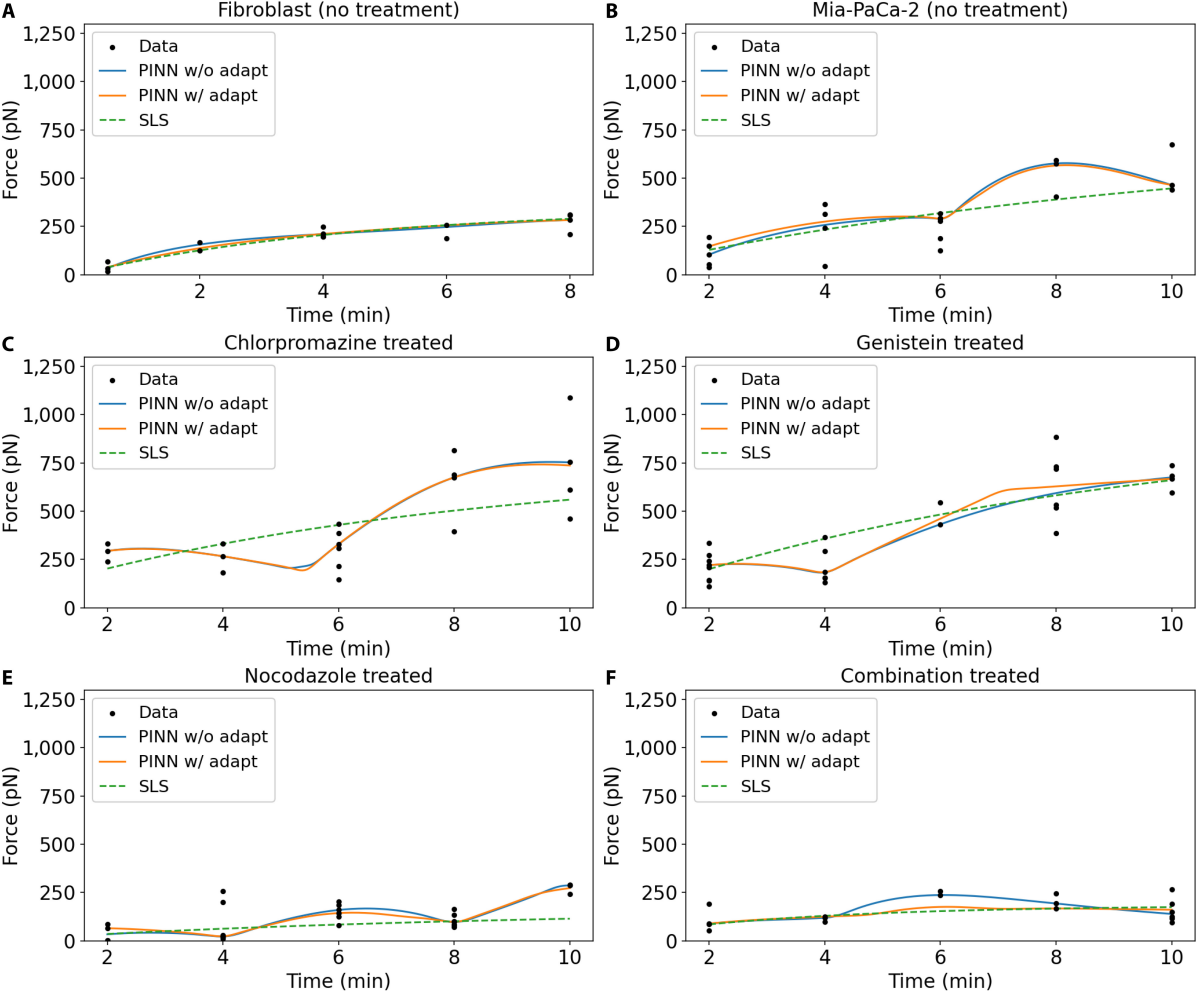
Comparative model fits across cell types and treatments. It compares the performance of 3 models, the classical viscoelastic SLS model, a Physics-Informed Neural Network (SLS_PINN) without adaptive weights, and an SLS_PINN with adaptive loss weights, in fitting force–time data across 6 experimental conditions. Panels (A) and (B) represent untreated and pharmacologically treated fibroblasts and MiaPaCa-2 cells. Panels (C) to (F) represent the MiaPaCa-2 cells treated with Chlorpromazine, Genistein, Nocodazole, and the combined 3 inhibitors, respectively. Black dots correspond to experimental force measurements at discrete adhesion durations (0.5 to 10 min) for untreated fibroblast and (2 to 10 min) for the untreated and the treated MiaPaCa-2 cells.

Figure 6A shows model fits for force–time data from untreated fibroblasts, where force increases smoothly from ~39 pN at *t* = 0.5 min to ~280 pN at *t* = 8 min with modest variance. All 3 models closely follow the data and are visually nearly indistinguishable, with only a slight early-time deviation where the SLS curve lies marginally below the PINN variants. Consistent with this observation, the SLS model achieves LOO-*R*^2^ = 0.812 ± 0.142. The nonadaptive SLS_PINN yields $\mathrm{LOO}-R^{2}=0.742\pm 0.207$ ($\mathrm{\Delta LOO}-R^{2}=-0.070$), with training behavior shown in the top panel of Fig. S3B. The remaining metrics favor the SLS model, with LOO-LL decreasing from −76.18 to −78.57 and LOO-RMSE increasing from 38.85 to 45.56 pN, indicating that the neural correction does not improve predictive performance. Consistent with this, the neural correction (Fig. S3C) is large and oscillatory (dotted line), peaking near +30 pN at ~2 min before dropping to ~−10 pN, suggesting overfitting. The adaptive SLS_PINN produces nearly identical predictive metrics (LOO-*R*^2^ = 0.742 ± 0.198, LOO-LL = −78.57, LOO-RMSE = 45.57 pN) as shown in the bottom panel of Fig. S3B. However, the training dynamics differ substantially: the physics weight increases strongly (1/σ_p_ ≈ 1 → 10), while the data weight decreases ($1/\sigma_{d}\approx 1\to 0.1$). As a result, the adaptive neural correction (Fig. S3C, solid green) is smaller and smoother, peaking at ~12 pN. Overall, the consistent underperformance of both PINN variants indicates that untreated fibroblast adhesion is already well captured by the SLS model, and the neural correction provides no additional benefit.

#### Chlorpromazine-treated MiaPaCa-2 cells

Chlorpromazine, an inhibitor of clathrin-mediated endocytosis, produces highly variable adhesion dynamics that challenge all models (Fig. 6C). The SLS model (green dashed) predicts a near-linear force increase reaching ~570 pN at *t* = 10 min and fails to capture the sigmoidal mid-phase acceleration seen in the data. In contrast, both PINN variants reproduce the sigmoidal trajectory, rising steeply to ~760 pN at *t* ≈ 8 min before a slight decline, and are visually nearly identical. The SLS model yields LOO-*R*^2^ = 0.160 ± 0.110, reflecting poor fit to the nonlinear force behavior. The nonadaptive SLS_PINN increases LOO-*R*^2^ to 0.501 ± 0.211 (ΔLOO-*R*^2^ = +0.341), as also reflected in the training loss (Fig. S4B). More robust improvements appear in the remaining metrics: LOO-LL improves from −136.33 to −131.12 (ΔLOO-LL = +5.21) and LOO-RMSE decreases from 220.82 to 170.25 pN (Δ = −50.57 pN), indicating substantially better out-of-sample performance. The neural correction term (Fig. S4C) explains this improvement, showing a large negative trough (~−150 pN at *t* ≈ 5 to 6 min) followed by strong positive recovery (~+175 to 220 pN at *t* ≈ 8 to 9 min), effectively reshaping the SLS backbone to match the sigmoidal force trajectory. Training dynamics (Fig. S4B) show rapid loss reduction with data loss dominating and physics loss near zero, indicating that the model prioritizes fitting the irregular data. The adaptive SLS_PINN produces slightly lower performance (LOO-*R*^2^ = 0.449 ± 0.202; LOO-RMSE = 178.83 pN; ΔLOO-LL = −0.99 relative to the nonadaptive model), as shown in Fig. S4C and D. The adaptive weights (Fig. S4D) show moderate reweighting ($1/\sigma_{p}$ ≈ 1 → 1.35; $1/\sigma_{d}\approx$ 1 → 0.12), which suppresses the neural correction and slightly reduces predictive performance in this condition where large data-driven corrections are required. Biological factors such as altered membrane tension [63] and modulation of integrin activity [64] further amplify measurement variability, as may compensatory clathrin-independent pinocytosis [43,44] and enhanced integrin clustering under clathrin blockade [65]. These biological complexities collectively render this condition one where no current modeling framework achieves reliable predictive performance.

#### Genistein-treated MiaPaCa-2 cells

Genistein, an inhibitor of caveolae-mediated endocytosis, perturbs a different uptake pathway than Chlorpromazine and produces moderate disruption to adhesion mechanics. Figure 6D shows that the SLS model fails to capture the nonmonotonic force trajectory, predicting a linear increase while missing the mid-phase force dip at *t* ≈ 4 min, where forces fall to ~150 pN before recovering to ~650 pN at *t* = 10 min. Both PINN variants reproduce this local minimum and subsequent recovery, tracking the data closely and producing nearly identical curves. The SLS model achieves LOO-*R*^2^ = 0.601 ± 0.073. The nonadaptive SLS_PINN increases this slightly to LOO-*R*^2^ = 0.633 ± 0.136 (ΔLOO-*R*^2^ = +0.032), as reflected in the training loss (Fig. S5B, top panel). However, this difference is smaller than the associated standard deviation and therefore not meaningful based on LOO-*R*^2^ alone. LOO-LL improves modestly from −166.45 to −165.39 (ΔLOO-LL = +1.06) and LOO-RMSE decreases from 145.92 to 140.11 pN (Δ = −5.81 pN), indicating only weak improvement over SLS. The neural correction (Fig. S5C) explains this behavior: both corrections show a pronounced negative trough (~−170 pN at *t* ≈ 4 min), matching the observed force minimum, followed by recovery to ~+50 pN at *t* ≈ 8 min. Training dynamics (Fig. S5B) show rapid loss reduction with data loss dominating and physics loss near zero. The adaptive SLS_PINN produces very similar performance (LOO*-R*^2^ = 0.639 ± 0.130; LOO-LL = −165.15; LOO-RMSE = 138.81 pN), with negligible differences relative to the nonadaptive model (Δ*R*^2^ = +0.006, ΔLOO-LL = +0.24, ΔLOO-RMSE = −1.30 pN). The adaptive weights (Fig. S5D) show moderate reweighting (1/σ_p;_ ≈ 1 → 1.85; $1/\sigma_{d}$ ≈ 1 → 0.12), but this does not translate into measurable predictive improvement. The adaptive loss converges slightly lower than the nonadaptive variant (Fig. S5B), indicating improved training stability even in the absence of a measurable out-of-sample performance gain. Sustained high adhesion forces despite caveolar inhibition suggest that alternative uptake routes such as micropinocytosis maintain strong, persistent membrane–particle interactions [66,67]. The small but consistent LOO-RMSE improvements shared by both PINN variants over SLS warrant cautious optimism, though the small magnitude and partially overlapping LOO-*R*^2^ confidence intervals prevent a definitive conclusion of genuine mechanistic improvement over the SLS baseline.

#### Nocodazole-treated MiaPaCa-2 cells

Figure 6E shows modeling results for MiaPaCa-2 cells treated with Nocodazole. It reveals the strongest qualitative divergence between the SLS model and the PINN variants across all conditions. The SLS model predicts a near-flat trajectory below ~100 pN and fails to capture the complex nonmonotonic force dynamics. In contrast, both PINN variants reproduce the main structural features: a local minimum at *t* ≈ 4 min, recovery to ~160 pN at *t* ≈ 6 min, a secondary dip near *t* ≈ 7 to 8 min, and a late rise to ~250 to 280 pN at *t* = 10 min. The SLS model yields LOO-*R*^2^ = 0.055 ± 0.117, indicating very poor fit. The nonadaptive SLS_PINN increases this to LOO-*R*^2^ = 0.396 ± 0.285 (ΔLOO-*R*^2^ = +0.341). Although the large standard deviation limits interpretation of *R*^2^, other metrics show clear improvement: LOO-LL increases by ΔLOO-LL = +6.94 (−180.99 → −174.05) and LOO-RMSE decreases from 83.05 to 66.40 pN (Δ = −16.65 pN), indicating better predictive performance. The neural correction (Fig. S6C, dotted line) follows a strongly oscillatory trajectory, reaching ~+70 pN at *t* ≈ 6 min and rising to ~+145 pN at *t* = 10 min, reflecting large adjustments to the SLS backbone. The adaptive SLS_PINN produces similar predictive performance (LOO-*R*^2^ = 0.371 ± 0.280; ΔLOO-*R*^2^ = −0.025; ΔLOO-LL = −0.38; ΔLOO-RMSE = +1.33 pN relative to the nonadaptive model). However, the adaptive weight dynamics (Fig. S6D) show a distinct 2-phase pattern: 1/σ_p_ initially decreases (~1 → 0.3) before gradually increasing to ~1.15 by epoch 8,000, while $1/\sigma_{d}$ declines to ~0.1. This behavior suggests early emphasis on data fitting followed by increasing physical regularization. Despite this reweighting, both neural corrections remain similar in magnitude and shape (Fig. S6C), indicating that the complex force trajectory requires a large data-driven correction regardless of weighting strategy. Biologically, the low and variable adhesion forces likely reflect mechanical decoupling of the plasma membrane from intracellular structures following cytoskeletal disruption, while the late-phase force recovery captured by both PINN variants may reflect residual actin-based adhesion that persists independently of microtubule integrity [68].

#### Combination-treated MiaPaCa-2 cells

Figure 6F shows results for the most severe perturbation tested, combining inhibition of clathrin- and caveolae-mediated endocytosis with microtubule depolymerization. The SLS model yields LOO-*R*^2^ = 0.030 ± 0.273, and LOO-LL = −99.97. The nonadaptive SLS_PINN produces LOO-*R*^2^ = 0.257 ± 0.318, though *R*^2^ remains unreliable due to large uncertainty. Other metrics indicate modest improvement: LOO-LL adjusts to −97.58 and LOO-RMSE decreases from 62.49 pN to 54.70 pN (Δ = −7.79 pN), suggesting that the neural correction captures limited residual structure. However, the correction (Fig. S7C, dotted line) oscillates between ~+85 and −25 pN, amplitudes comparable to the signal itself, indicating partial noise fitting. The adaptive SLS_PINN shows nearly identical predictive performance (ΔLOO-*R*^2^ = +0.015, ΔLOO-LL = +0.19, ΔLOO-RMSE = −0.55 pN) but through different training dynamics. The physics weight 1/σ_p_ increases sharply from ~4 to ~10 between epochs 4,000 and 5,500 (Fig. S7D), while the adaptive neural correction (Fig. S7C, solid) is much smaller and smoother, peaking near ~20 pN. This suppression indicates that the adaptive mechanism recognizes that large corrections are not justified by the noisy data, producing a more physically interpretable fit despite similar predictive accuracy. Biologically, simultaneous blockade of 2 major endocytic routes alongside microtubule depolymerization abolishes coordinated force transmission, leaving only fleeting localized membrane rearrangements that render standard continuum models fundamentally inadequate for this condition [69,70]. These interpretations remain phenomenological because the present framework models force dynamics without directly measuring receptor density, cytoskeletal organization, or signaling pathways.

**Fig. 7.**
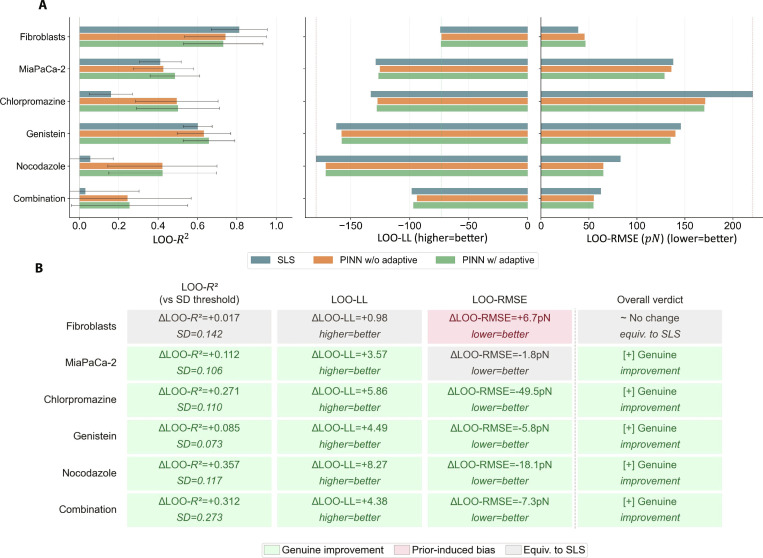
(A) Multimetric performance evaluation of SLS and SLS_PINN models across 6 experimental conditions. Left: LOO-*R*^2^ ± SD as grouped bars (3 models × 6 conditions) with error bars showing the SD from LOO resampling, making the SD-threshold interpretation visually immediate. Center: LOO-LL, scaled so longer bar = better fit. Right: LOO-RMSE, where the Chlorpromazine and Genistein bars visually expose the overfitting pattern (PINN bars longer = worse, despite LOO-*R*^2^ appearing to improve). (B) Condition × metric classification matrix: stabilization vs. prior-induced bias: Each cell classifies the outcome of adaptive SLS_PINN weighting relative to the SLS baseline for a given condition and metric.

Figure 7 provides a cross-condition overview of all 3 frameworks across 6 experimental conditions. The three-metric bar chart (Fig. 7A) and the metric summary (Fig. 7B) together reveal patterns that would not be apparent from any single metric in isolation. The SLS model provides adequate fits only for untreated fibroblasts, where both PINN variants offer no generalization improvement (ΔLOO-RMSE = +6.7 pN). Across all 5 MiaPaCa-2 conditions, both PINN variants achieve genuine multimetric improvements over SLS, with the largest LOO-RMSE gains under Chlorpromazine (Δ = −50.6 pN) and Nocodazole (Δ = −16.7 pN) treatment. The bar chart illustrates that *R*^2^ confidence intervals overlap substantially across conditions, while LOO-LL and LOO-RMSE provide cleaner model separation, reinforcing the importance of multimetric evaluation. The adaptive and nonadaptive PINN variants are statistically equivalent in out-of-sample performance across all 6 conditions, with the primary advantage of the adaptive variant lying in producing smaller, physically regularized neural corrections that are less contaminated by noise-driven overfitting. These results indicate that the adaptive SLS_PINN is the preferred framework for cross-condition phenotyping, providing physically consistent parameter estimates across the full spectrum of cytoskeletal perturbation severity studied here.

### Predictive performance and uncertainty quantification of the physics-informed Bayesian models for adhesion kinetics across perturbed and control cell conditions

To develop a robust data-driven model that integrates physical constraints with uncertainty quantification, we designed a hybrid framework based on physics-informed Bayesian inference with an SLS prior (see the pseudocode of the training algorithm in Fig. 4). This approach models the time evolution of the extraction force during nanoparticle–cell membrane adhesion by leveraging the predictive structure of the SLS model as a prior, combined with a heteroscedastic Bayesian likelihood, as presented in the “Physics-informed Bayesian modeling for adhesion kinetics” section.

The heteroscedastic noise model, as presented in Eq. (9), is partially constructed from a smoothed empirical estimate of the local standard deviation of the observed data, rather than being fully specified as a latent stochastic process with an associated prior. This empirical component is combined with inferred scaling parameters within the Bayesian framework, yielding an empirical Bayes-type formulation of heteroscedasticity. Although this does not constitute a fully Bayesian treatment of the noise process, it provides a stable and data-adaptive approximation of the variance structure, particularly under conditions of limited and uneven temporal sampling, as in the present study. Consequently, the inferred uncertainty should be interpreted as conditional on this empirical variance estimate.

**Fig. 8.**
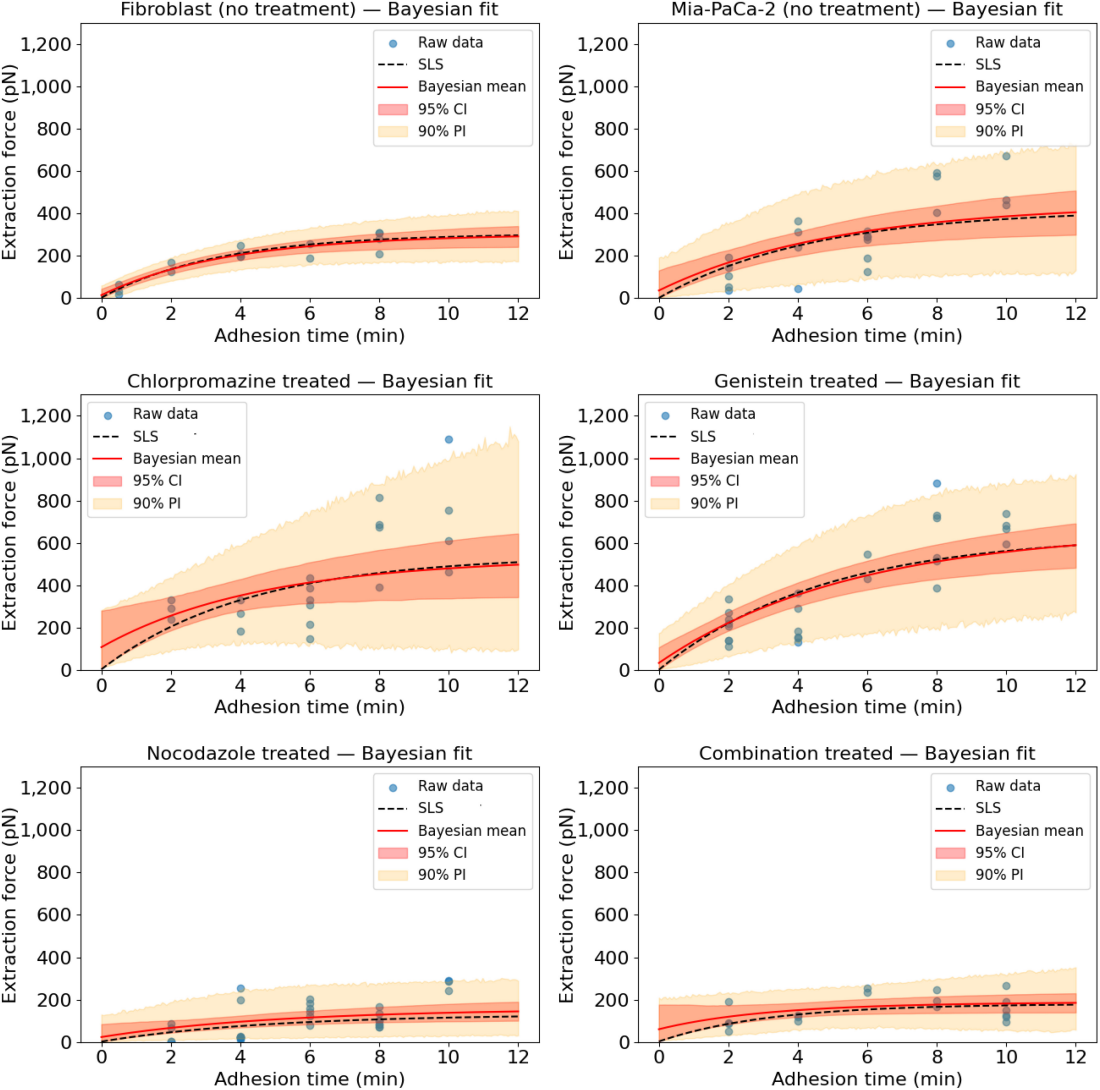
Posterior distributions of adhesion force across biological conditions using the hybrid PINN–Bayesian model. Each panel shows observed force measurements (blue dots), the Bayesian posterior mean (red curve), 90% prediction interval (light orange), and 95% credible interval (dark red). Dashed black lines represent the SLS prior generated before Bayesian updating. Conditions include untreated fibroblast cells, untreated pancreatic cancer cells (MiaPaCa-2), and MiaPaCa-2 cells treated with inhibitors: Chlorpromazine, Genistein, Nocodazole, and a combination of all 3 inhibitors. The Bayesian model accurately captures force dynamics and uncertainty under both control and perturbed conditions. The empirical coverage of the 90% posterior predictive interval across all conditions is 93.9% (123/131 observations), consistent with the nominal 90% level and confirming that the Bayesian model is well-calibrated.

Figure 8 illustrates the model’s performance across 6 experimental conditions: untreated fibroblast cells, untreated MiaPaCa-2 cells, and MiaPaCa-2 cells treated with Chlorpromazine, Genistein, Nocodazole, and a combination of all 3 inhibitors. Each subplot presents the raw experimental force data (blue dots), the SLS-based PI prior prediction (black dashed line), the Bayesian posterior mean (red line), the 95% credible interval (shaded red), and the 90% prediction interval (shaded yellow). The 90% posterior predictive interval serves as a posterior predictive check, confirming that the observed data points fall within the model’s predictive distribution across all 6 conditions, indicating that the Bayesian model is well-calibrated with respect to the observed measurements. These visualizations highlight the model’s fidelity, the propagation of uncertainty, and the biological relevance under varying cellular conditions.

In untreated fibroblasts, the SLS model and Bayesian posterior are closely aligned across the adhesion timeline, with narrow 95% CI (typically ±50 pN) and 90% PI (±100 pN). The force trajectory shows a smooth nonlinear increase, reaching about 400 pN at 10 min. These tight uncertainty bounds and close tracking of data (blue dots) suggest minimal uncertainty and strong physical agreement, confirming that untreated fibroblasts constitute the most linear and physically consistent system in the dataset.

In untreated MiaPaCa-2 pancreatic cancer cells, a similar trend is observed, though the 90% PI widens substantially after 6 min (up to ±250 to 300 pN), especially as extraction forces exceed 500 pN at 10 min. The deviation between the prior and posterior increases slightly, indicating higher heterogeneity in cancer cell adhesion dynamics. Despite this, the 95% CI remains narrow (within ±75 pN), reflecting that while the biological variability is higher, the model can still provide reliable and interpretable predictions.

In Chlorpromazine-treated MiaPaCa-2 cells, the Bayesian posterior initially shows a noticeable gap from the SLS prior, which narrows around 3 to 4 min. However, this is followed by a progressively widening divergence beyond 6 min, indicating increased deviation from the expected physical behavior as adhesion progresses. The force trajectory becomes more variable, ranging from ~100 to >700 pN, and the prediction interval widens to ±400 pN in later stages. This increased uncertainty is consistent with the reported disruption of clathrin-mediated endocytosis by Chlorpromazine, which may alter membrane mechanics and contribute to more heterogeneous adhesion force dynamics. The 95% CI, broader than in previous cases (often exceeding ±150 pN), reflects less confident parameter inference. Still, the Bayesian mean captures the upward force trend, showing the model’s resilience under moderate perturbations.

For Genistein-treated cells, the SLS model and Bayesian posterior remain closely aligned across the entire adhesion timeline, with only minor deviations between 2 and 6 min. Extraction forces peak at ~600 pN by 10 min, and the 90% PI remains relatively narrow (within ±150 to 200 pN), similar to the untreated fibroblast condition. The 95% CI is also compact, generally staying below ±80 pN, indicating that the model retains high certainty. These results suggest that caveolar inhibition via Genistein induces only mild nonlinearities and preserves most of the system’s viscoelastic characteristics.

In the Nocodazole-treated condition, the measured adhesion forces remain low (<200 pN). The Bayesian posterior initially exhibits a notable divergence from the SLS model, but this gap gradually narrows around 4 to 6 min. Meanwhile, the 90% prediction interval expands considerably, reaching widths of approximately ±250 to 300 pN, reflecting high uncertainty and increased variability in the force measurements. The 95% CI widens as well (up to ±100 pN), especially in late adhesion phases. These features may reflect the increased mechanical variability expected after microtubule disruption, together with the lower signal-to-noise ratio of the measured force trajectories. The model effectively communicates that reliable trend inference is difficult under such perturbation.

The combination treatment (Chlorpromazine, Genistein, and Nocodazole) leads to the most disrupted condition. Extraction forces remain low and irregular (mostly <200 pN) with a small increasing trend, and the SLS prior is nearly horizontal with a small increasing trend. The posterior mean is similarly near flat, while the 90% PI reaches its widest values, often exceeding ± 400 to 500 pN early in the timeline. The 95% CI also broadens (often >±150 pN), indicating maximal aleatoric uncertainty. This divergence suggests that the model cannot confidently identify trends and instead relies on its physical priors to avoid overfitting. This behavior is consistent with the absence of a clear force trend in this highly perturbed condition, where cytoskeletal and membrane processes are strongly disrupted.

This result demonstrates that the physics-informed Bayesian modeling for adhesion kinetics not only models adhesion force dynamics but also communicates confidence in its predictions. In simple, linear systems (e.g., fibroblasts, untreated MiaPaCa-2 cells, and Genistein-treated cells), the model provides accurate and low-uncertainty estimates. As system complexity increases due to endocytic or cytoskeletal perturbations (e.g., Chlorpromazine, Nocodazole, and combination), the model expresses higher uncertainty through wider confidence and prediction intervals and diverging posteriors. This shift likely reflects increased biological variability and potential model mismatch. Rather than overfitting noisy data, the physics-informed Bayesian architecture transparently communicates the limits of inference inherent in sparse datasets, making it especially suitable for modeling noisy, heterogeneous biological systems with interpretable uncertainty. This ensures that parameters $F_{0},$ $F_{\max},$ and τ are interpreted as effective kinetic phenotypes describing adhesion force dynamics, particularly in regimes where the mono-exponential assumption may simplify the underlying biological complexity. The empirical coverage of the 90% posterior predictive interval across all conditions is 93.9% (123/131 observations), consistent with the nominal 90% level and confirming that the Bayesian model is well-calibrated. The Nocodazole condition shows lower coverage (87.1%), consistent with the structural limitations of the mono-exponential SLS backbone under bimodal force distributions in this condition.

### Inference of kinetic parameters from physics-informed Bayesian modeling across perturbed adhesion pathways

The posterior distributions reported in this section were estimated from 2,000 pooled samples across 4 independent chains, with $\hat{R}$ ≤ 1.01 and ${\mathrm{ESS}}_{\text{bulk}}$ > 400 confirmed for all parameters and conditions (Fig. S14), ensuring that the inferred kinetic parameters reflect reliable and well-converged posterior estimates.

**Fig. 9.**
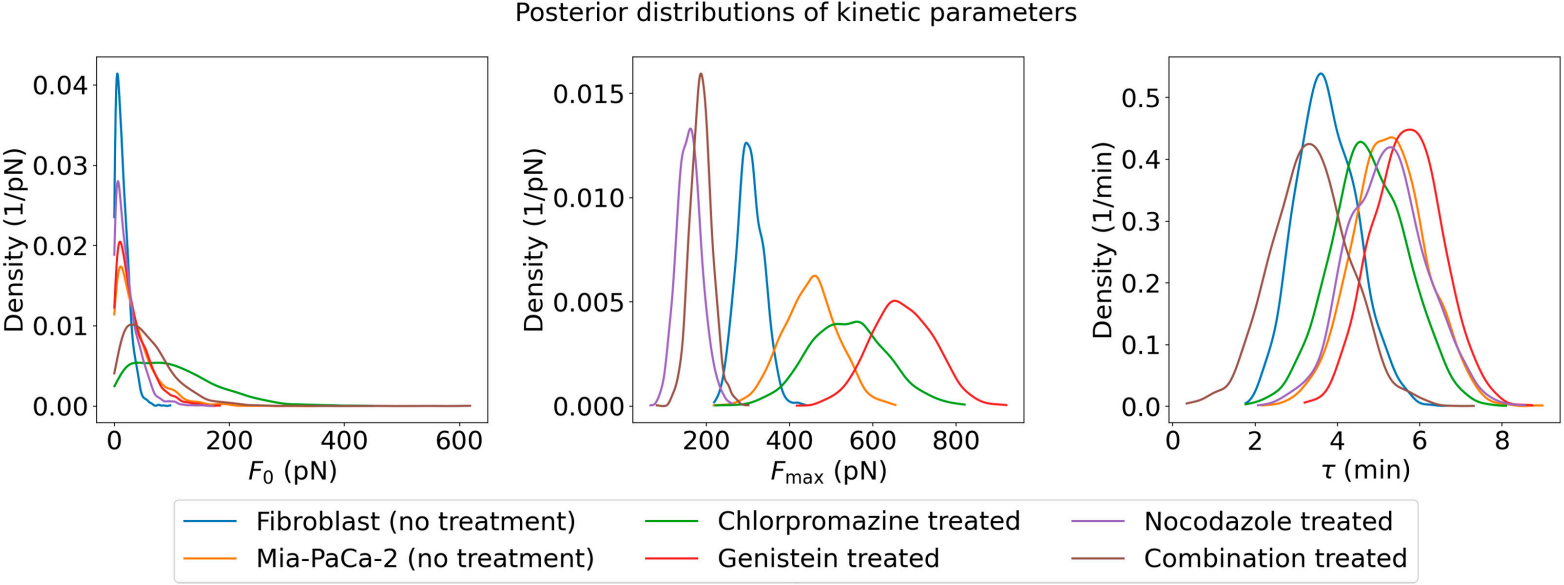
Posterior distributions of kinetic adhesion parameters inferred by the PINN–Bayesian model across 6 biological conditions. Each panel shows the kernel density estimate (KDE) of the marginal posterior distribution for (left) the initial effective contact force $F_{0}$ (pN), (center) the effective maximum adhesion force $F_{\max}$(pN), and (right) the effective characteristic adhesion time constant τ (min). Distributions were estimated from 2,000 pooled posterior samples (4 independent chains × 500 draws per chain) after confirming convergence via R-hat < 1.01 and ESS > 400 for all parameters across all conditions (Fig. S14). Parameters are interpreted as effective kinetic phenotypes of the collective adhesion behavior rather than direct measures of isolated molecular processes.

To characterize adhesion dynamics underlying the experimental force–time data, we analyzed the posterior distributions of 3 key kinetic parameters inferred through the physics-informed Bayesian framework: the initial effective contact force $F_{0}$, the effective final adhesion force $F_{\max}$, and the effective characteristic adhesion time constant τ. While these parameters provide a quantitative description of the adhesion process, they are interpreted as effective kinetic phenotypes, mathematical representations of the collective adhesion behavior, rather than direct measures of isolated biological events, given the model’s reliance on a mono-exponential backbone. These parameters were estimated for each of the 6 biological conditions under study: untreated fibroblast cells, untreated pancreatic cancer cells (MiaPaCa-2), and MiaPaCa-2 cells treated with inhibitors Chlorpromazine, Genistein, Nocodazole, and a combination of all 3 inhibitors, as depicted in Fig. 9.

The posterior distributions of the initial effective adhesion force $F_{0}$ vary notably across conditions: untreated fibroblasts and Nocodazole-treated cells exhibit the sharpest, narrowest peaks close to zero (<20 pN), indicating consistently weak and homogeneous initial adhesion; untreated MiaPaCa-2 cells and Genistein-treated cells show nearly identical, moderately broader distributions, suggesting that inhibition of caveolae-mediated endocytosis does not substantially alter initial contact force relative to the untreated state; Chlorpromazine-treated cells display a broader, more right-shifted distribution well beyond 300 pN, reflecting greater heterogeneity in initial adhesion; while combination-treated cells show the broadest distribution with the longest tail extending well beyond 200 pN, indicating the greatest variability and strongest instances of initial adhesion force across all conditions.

The posterior distributions of the effective maximum adhesion force $F_{\max}$ reveal distinct trends linked to cell type and treatment applied. Nocodazole-treated cells display the sharpest and narrowest peak at the lowest force level (~150 pN), indicating that microtubule disruption most severely constrains the adhesion plateau and reduces inter-replicate variability. Combination-treated cells show a similarly sharp and narrow peak marginally to the right (~180 pN), suggesting that simultaneous multipathway inhibition produces a comparable but slightly less severe constraint on maximum adhesion force. Fibroblasts display a sharp narrow peak at ~300 pN, reflecting a relatively uniform and moderate adhesion plateau characteristic of this cell type. In contrast, untreated MiaPaCa-2 cells exhibit a broader distribution centered around ~450 pN, indicating more heterogeneous and generally stronger adhesion. This broadening and rightward shift is further amplified in Chlorpromazine-treated cells (~500 to 550 pN) and most prominently in Genistein-treated cells, which display the broadest distribution centered around ~650 pN with a tail extending to ~850 pN. These shifts in $F_{\max}$ suggest that while certain treatments alter the observed adhesion capacity, these changes likely reflect the net result of complex cellular remodeling rather than a single causal change in molecular binding. Overall, $F_{\max}$ distributions reflect the interplay between cell type, treatment effects, and the biophysical mechanisms regulating effective adhesion strength.

The posterior distributions of the effective adhesion time constant τ reveal important differences among cell types and treatments. Untreated fibroblasts show a peak around 3.7 min with a range of about 1.7 to 6 min, indicating more consistent and relatively faster adhesion dynamics. Untreated MiaPaCa-2 pancreatic cancer cells have a peak shifted to about 5.2 min with a spread around 2.5 to 8 min, suggesting slower effective adhesion times compared to fibroblasts. Nocodazole results in a peak near 5.1 min, almost overlapping with the untreated Mia-PaCa-2 baseline. Chlorpromazine shifts the peak toward faster kinetics at 4.2 min, while the combination treatment shows the fastest profile of all treated groups, peaking at ~3.2 min. Conversely, Genistein is the only treatment that prolongs the process, with a peak shifted rightward to 6 min. While most groups show a similar overall width, the fibroblasts and the combination-treated cells exhibit slightly narrower distributions. This suggests a marginally more uniform kinetic response in these groups compared to the more heterogeneous profiles of the untreated or Nocodazole-treated cancer cells. In sum, both cell type and inhibitor treatments modulate the temporal profile of cell adhesion, with cancer cells and treated cells generally exhibiting longer and more variable adhesion times compared to untreated fibroblasts.

In summary, the physics-informed Bayesian analysis of the key kinetic parameters $F_{0}$, $F_{\max}$, and τ reveals condition-dependent differences in inferred adhesion dynamics across cell types and treatment conditions that are consistent with distinct underlying phenotypes, though the mono-exponential model structure precludes attribution to specific molecular mechanisms. The variations in effective initial and maximum adhesion forces, along with the prolonged and more heterogeneous effective adhesion time constants under inhibitor treatments, are consistent with pharmacological modulation of both the strength and temporal behavior of cell adhesion, as captured by the model’s effective parameters. These findings provide quantitative descriptors of the biophysical effects of different treatments on cellular adhesion and illustrate the value of combining mechanistic modeling with probabilistic inference as a phenotyping strategy for complex biological processes, while noting that causal mechanistic interpretation requires complementary experimental verification.

To assess dependence on prior specification, we performed a sensitivity analysis repeating Bayesian inference under 3 prior-width regimes spanning a 12-fold range: narrow ($\sigma_{F_{\max}^{*}}$, $\sigma_{F_{0}^{*}}$ = 50 pN, $\sigma_{\tau^{*}}\;$ = 0.5 min), original ($\sigma_{F_{\max}^{*}}$, $\sigma_{F_{0}^{*}}$ = 300 pN, $\sigma_{\tau^{*}}$ = 1 min), and wide ($\sigma_{F_{\max}^{*}}$, $\sigma_{F_{0}^{*}}$ = 600 pN, $\sigma_{\tau^{*}}$ = 3 min). The percentage shift in posterior mean relative to the original prior is shown in Fig. S15. Under the narrow prior, all parameters remained robust across all 6 conditions, with shifts below 12% for $F_{\max}$ and *τ* and below 34% for $F_{0}$ (the larger percentage for $F_{0}$ reflects its small absolute magnitude rather than biological implications). Under the wide prior, *τ* showed shifts of +25% to +56% in conditions with sparser replication at certain time points (Mia-PaCa-2, Genistein, Nocodazole, and Chlorpromazine), while $F_{\max}$ showed moderate shifts of +6% to +29% in the same conditions. $F_{0}$ remained stable under the wide prior (shifts ≤ +45% in absolute percentage).

Comparison of prior and posterior distributions (Fig. S16) confirms likelihood-dominated inference for $F_{0}$, $F_{\max}$, and *τ* across all 6 conditions: the posteriors are dramatically narrower than the priors, spanning less than 5% of the prior range. For *τ*, the posterior is moderately narrower than the prior, consistent with weaker identifiability of the time constant near the observation window boundary (*t* ≤ 10 min). When *τ* approaches or exceeds the observation window, the exponential saturation curve is not fully resolved in the data, and the likelihood cannot strongly constrain *τ*, allowing a wider prior to pull the posterior upward. The sensitivity analysis identified *τ* as the parameter most susceptible to prior influence in conditions with sparse replication at time points, attributable to the finite observation window (*t* ≤ 10 min). The original prior width ($\sigma_{F_{\max}^{*}}$, $\sigma_{F_{0}^{*}}$ = 300 pN, $\sigma_{\tau^{*}}$ = 1 min) therefore represents a principled and conservative choice balancing broad exploration with physical plausibility. The sensitivity analysis identified *τ* as the parameter most susceptible to prior influence in conditions with sparse replication at time points, attributable to the finite observation window (*t* ≤ 10 min).

To further characterize the posterior geometry of the inferred kinetic parameters and exploit a key advantage of the Bayesian framework, we examined the pairwise joint posterior distributions of $F_{0}$, $F_{\max}$, and *τ* across all 6 experimental conditions (Fig. S17). The $F_{\max}$ and *τ* correlation emerges as the dominant and most consistent structural feature across conditions: it is strongly positive in untreated fibroblast (Pearson correlation coefficient *r* = +0.77) and untreated MiaPaCa-2 cells (*r* = +0.56), and remains notably positive in Genistein-treated cells (*r* = +0.63), indicating that conditions with a higher effective adhesion plateau also tend to exhibit a longer characteristic adhesion timescale. In contrast, the $F_{0}$ and $F_{\max}$ correlation is consistently negative across all conditions (*r* ranging from −0.23 to −0.42), with the strongest anti-correlation observed in Chlorpromazine-treated cells (*r* = −0.42), suggesting a compensatory posterior relationship between the initial contact force and the adhesion plateau that is amplified under pharmacological membrane perturbation. Notably, the $F_{0}$ and *τ* correlation is negligible in all conditions (|*r*| ≤ 0.21), confirming that the initial effective contact force and the adhesion timescale are effectively orthogonal dimensions of the posterior and that the 3-parameter model does not suffer from a global identifiability collapse. Taken together, these results demonstrate that the physics-informed Bayesian framework not only yields marginal parameter estimates but also resolves the full joint posterior structure, revealing that the $F_{\max}$ and *τ* posterior dependence constitute the primary source of residual posterior uncertainty and that pharmacological perturbation selectively modulates the $F_{0}$ and $F_{\max}$ dependence without substantially altering the independence of the timescale parameter.

## Conclusion

In this study, we addressed these challenges by developing a hybrid PINN framework that captures early-stage adhesion kinetics. By integrating an analytical SLS model as a mechanistic backbone to represent baseline viscoelasticity, and augmenting it with a neural residual term to account for nonlinear and stochastic deviations, we created a model that is both physically grounded and highly adaptive.

Our methodology advances robust parameter inference through the inclusion of an adaptive, uncertainty-aware loss weighting scheme and a physics-informed Bayesian framework. By utilizing a heteroscedastic likelihood, the model effectively navigates the heterogeneity of experimental data across different cell types and pharmacological treatments. Validation via leave-one-out cross-validation confirmed that this hybrid approach provides condition-dependent predictive improvements over classical models, particularly in biological states where cytoskeletal perturbations make the adhesion process highly irregular and where the SLS backbone alone is insufficient to capture the observed variability.

Ultimately, this work presents an interpretable framework for cross-condition phenotyping, providing physically consistent parameter estimates across a diverse spectrum of biological environments. By bridging the gap between data-driven machine learning and mechanistic biophysics, this approach suggests a potentially generalizable strategy for analyzing noisy, heterogeneous biological systems. It provides a foundation for future research into targeted drug delivery and mechanobiology, where understanding the time-dependent evolution of adhesion strength may inform, but not alone determine, predictions of cellular uptake and therapeutic efficacy.

## Data Availability

The data that support the findings of this study are available from the corresponding author (H.B.) upon request.

## Supplementary Materials

Supplementary 1 Supplementary Notes Figs. S1 to S17
