# Supplementary material for: Hybrid Physics-Informed and Bayesian Modeling of Single-Nanoparticle–Cell Adhesion Kinetics under Cytoskeletal Perturbation
Source: Comput Struct Biotechnol J. 2026 May 11;35(1):0077. doi: 10.34133/csbj.0077 (PMC13158457; doi:10.34133/csbj.0077)
Supplement: Supplementary 1 — Supplementary Notes Figs. S1 to S17 [file csbj.0077.f1.docx]

**Supporting information**

**Hybrid Physics-Informed and Bayesian Modeling of Single-Nanoparticle–Cell Adhesion Kinetics under Cytoskeletal Perturbation**

*Houari Bettahar ^a,^ *, Hélder A. Santos ^b, c,^, Quan Zhou ^a,^*

*^a^ Department of Electrical Engineering and Automation, Aalto University, Maarintie 8, 02100 Espoo, Finland.*

*^b^ Drug Research Program, Division of Pharmaceutical Chemistry and Technology, Faculty of Pharmacy, University of Helsinki, FI-00014, Helsinki, Finland.*

*^c^ Department of Biomaterials and Biomedical Technology, The Personalized Medicine Research Institute (PRECISION), University Medical Center Groningen (UMCG), University of Groningen, 9713 AV, Groningen, the Netherlands.*

** Corresponding authors:* [*houari.bettahar@aalto.fi*](mailto:houari.bettahar@aalto.fi)

**Supplementary note**

**Figures S1-S17**

**Supplementary note**

**S.1 Viscoelastic modeling of time-dependent adhesion using the standard linear solid framework**

The process of nanoparticle adhesion to the cell membrane over time exhibits viscoelastic force development, where the adhesion force increases with longer contact durations.

To model this behavior, we employ the Standard Linear Solid (SLS) model, which captures both instantaneous elastic deformation and time-dependent viscous responses, key features of cellular mechanics during nanoparticle adhesion (see **Figure S1**). This approach is suited for receptor-independent internalization mechanisms, such as macropinocytosis, which dominate the uptake of larger nanoparticles (700–900 nm) used in our experiments [1] and do not involve specific receptor–ligand interactions [2]. Consequently, the model does not account for receptor–ligand binding kinetics, which are more relevant for the internalization of smaller nanoparticles (50–200 nm) [3–7]

Let $x\left( t \right)$denote the total deformation applied by the nanoparticle. This deformation is shared across two mechanical elements: a spring with stiffness $k_{1}$, and a Maxwell arm composed of a spring ($k_{2}$) and a dashpot ($\eta$) in series. These two branches are arranged in parallel, representing the combined elastic and viscoelastic behavior of the membrane-cytoplasm interaction. Let $x_{1}\left( t \right)$ denote the deformation in spring $k_{1}$, $x_{2}\left( t \right)$ the deformation in Maxwell arm (series spring + dashpot), and $F\left( t \right)$ the total force response.

Since $k_{1}$is in parallel with the Maxwell arm:

$$\begin{aligned} F\left( t \right)=k_{1}x_{1}\left( t \right)+F_{2}\left( t \right) \#\left( S1 \right) \end{aligned}$$

where $x_{1}\left( t \right)$ denote the deformation in spring $k_{1}$ and $F_{2}\left( t \right)$ is the force in the Maxwell arm.

$x_{2}\left( t \right)$ denotes the deformation in the Maxwell arm (series spring + dashpot):

$$\begin{aligned} x_{2}\left( t \right)=x_{s}\left( t \right)+x_{d}\left( t \right) \#\left( S2 \right) \end{aligned}$$

where $x_{s}\left( t \right)$ is the deformation in spring $k_{2}$ and $x_{d}\left( t \right)$ is deformation in dashpot $\eta$.

The force in the Maxwell arm is the same throughout:

$$\begin{aligned} F_{2}\left( t \right)= k_{2}x_{s}\left( t \right)=\eta\frac{dx_{d}}{dt}\#\left( S3 \right) \end{aligned}$$

Substitute into the previous equation:

$$\begin{aligned} \frac{dx}{dt}=\frac{1}{k_{2}}\frac{d\left[ F\left( t \right)-k_{1}x_{1}\left( t \right) \right]}{dt}+\frac{\left[ F\left( t \right)-k_{1}x_{1}\left( t \right) \right]}{\eta} \#\left( S4 \right) \end{aligned}$$

Multiply both sides by $k_{2}$​:

$$\begin{aligned} k_{2}\frac{dx}{dt}=\frac{dF}{dt}-k_{1}\frac{dx}{dt}+\frac{k_{2}}{\eta}F\left( t \right)- \frac{k_{1}k_{2}}{\eta}x\left( t \right) \#\left( S5 \right) \end{aligned}$$

Bring terms to one side:

$$\begin{aligned} \frac{dF}{dt}+\frac{k_{2}}{\eta}F\left( t \right)=\left( k_{1}+k_{2} \right)\frac{dx}{dt}+\frac{k_{1}k_{2}}{\eta}x\left( t \right) \#\left( S6 \right) \end{aligned}$$

For a constant deformation $\boldsymbol{x}\left( t \right)=X_{0} \left( i.e., \frac{dx}{dt}=0 \right)$, which reflects the experimental condition where the nanoparticle is held at a fixed contact before retraction, this simplifies to a first-order linear ordinary differential equation:

$$\begin{aligned} \frac{dF}{dt}+\frac{k_{2}}{\eta}F\left( t \right)=\frac{k_{1}k_{2}}{\eta}X_{0} \#\left( S7 \right) \end{aligned}$$

Then:

$$\begin{aligned} \frac{dF}{dt}+aF=aF_{max}\#\left( S8 \right) \end{aligned}$$

where the maximum force is $F_{max}= k_{1}X_{0}$, $a=\frac{k_{2}}{\eta}$ is the rate (inverse time) corresponding to a characteristic timescale $\tau=1/a$, and $F_{0}$is the initial contact force. This exponential formulation matches the observed saturation behavior in adhesion force measurements.

The analytical solution is:

$$\begin{aligned} F\left( t \right)=F_{0}+\left( F_{max}-F_{0} \right)\left( 1-e^{-at} \right)\#\left( S9 \right) \end{aligned}$$

**S.2 Probability Distributions**

**a- Normal distribution**

If $x\mathcal{\sim N}\left( \mu, \sigma\right)$, then $x$ is normally distributed with a mean $\mu$ and standard deviation $\sigma$. Its probability density function is:

$$\begin{aligned} p\left( x \mid\mu, \sigma\right)=\frac{1}{\sqrt{2\pi\sigma^{2}}}\exp\left( -\frac{\left( x-\mu\right)^{2}}{2\sigma^{2}} \right) \#(S10) \end{aligned}$$

**b- Truncated Normal distribution**

If $x\sim\mathcal{N}_{\geq0}\left( \mu, \sigma\right)$, then x follows a normal distribution truncated to non-negative values:

$$\begin{aligned} p\left( x \mid\mu,\sigma\right)=\left\{ \begin{aligned} \frac{1}{\sqrt{2\pi\sigma^{2}}}\exp\left( -\frac{\left( x-\mu\right)^{2}}{2\sigma^{2}} \right), &x\geq0 \\ 0, &x<0 \end{aligned} \right.\#(S11) \end{aligned}$$

**c- Half-Cauchy distribution:**

If $x\sim Half-Cauchy\left( \beta\right)$, then $x,$ follows a Half-Cauchy distribution with scale parameter $\beta$:

$$\begin{aligned} p\left( x \mid\beta\right)=\left\{ \begin{aligned} \frac{2}{\pi\beta\left( 1+\left( \frac{x}{\beta} \right)^{2} \right)}, &x\geq0 \\ 0, &x<0 \end{aligned} \right.\#\left( S12 \right) \end{aligned}$$

This prior is weakly informative, with heavy tails, and is commonly used for variance or scale parameters.

**S.3 Smooth L1 loss (Huber loss)**

For a prediction error $e =\hat{F}- F$, we use the Smooth L1 loss (also called the Huber loss), which is less sensitive to outliers than the standard squared error. It is defined as:

$$\begin{aligned} SmoothL1\left( e \right)=\left\{ \begin{aligned} \left( \frac{1}{2} \right)\cdot e^{2}, &\left| e \right|< 1 \\ e- \left( \frac{1}{2} \right), &\mathrm{otherwise} \end{aligned} \right.\#\left( S13 \right) \end{aligned}$$

This loss behaves like a squared error for small deviations ($\left| e \right|< 1$), and like a linear absolute error for large deviations $\left( \left| e \right|\geq1 \right)$, providing robustness to outliers while preserving smooth gradients for optimization.

**S.4 Performance estimation**

All metrics: the coefficient of determination (Loo-R² ± SD, where SD was estimated via leave-one-out resampling), leave-one-out resampling log-likelihood (LOO-LL), and leave-one-out root mean square error (LOO-RMSE) are computed using the full set of raw replicate measurements at each time point (4–5 replicates × 5 time points = 20–25 observations per condition), rather than averaging replicates within time points first. This ensures that replicate-level variability is fully represented in all performance estimates.

$\mathbf{LOO-R}^{\mathbf{2}}$ **and SD estimation:**

For each dataset, the coefficient of determination ($R^{2}$) is computed to assess how well the model predictions $\hat{F}_{i}$fit the observed data $F_{i}$. The R² is defined as:

$$R^{2}=1-\frac{\sum_{i=1}^{n} (F_{i}-\hat{F}_{i})^{2}}{\sum_{i=1}^{n} (F_{i}-F)^{2}}$$

where $n$ is the number of observations in the dataset, $\hat{F}_{i}$is the predicted value for the $i$-th observation, and $F$is the mean of all observed values.

To quantify the variability of the R² estimate, a leave-one-out (LOO) approach is applied: each observation is sequentially removed, and the model predictions are recalculated for the remaining $n-1$ points, and R² is recomputed. This produces a list of R² values $R_{-i}^{2}$for each leave-one-out iteration. The mean and standard deviation (SD) of these values are then calculated:

$${LOO-R}^{2}=\frac{1}{n}\sum_{i=1}^{n} R_{-i}^{2}, \text{SD }=\sqrt{\frac{1}{n-1}\sum_{i=1}^{n} \left( R_{-i}^{2} - {LOO-R}^{2} \right)^{2}}$$

These calculations are applied directly to the **r**aw replicate measurements at each time point, rather than averaging across time points first. This ensures that ${LOO-R}^{2}\pm SD$ reflects both the predictive stability of the model and the variability across replicates. The SD therefore directly quantifies metric instability arising from small sample size and replicate-level variability, including sensitivity to individual outlier observations. LOO-R² differences between models are treated as meaningful only when they exceed the larger of the two compared SDs; differences smaller than this threshold are treated as statistically equivalent regardless of their nominal size, since they cannot be distinguished from random variation due to limited replication.

**LOO-RMSE estimation**

For each dataset, the leave-one-out root mean square error (LOO-RMSE) is computed by iteratively leaving out one observation and fitting the model to the remaining $n-1$points. The predicted value for the left-out point is compared to the actual observation, and the RMSE is calculated as:

$$\text{LOO-RMSE}=\sqrt{\frac{1}{n}\sum_{i=1}^{n} \left( F_{i} - \hat{F}_{-i} \right)^{2}}$$

where $F_{i}$is the observed value of the $i$-th data point, and $\hat{F}_{-i}$is the model prediction for that point when it was excluded from the fit. This procedure captures the model’s predictive performance and sensitivity to individual data points. With N = 20–25 raw replicate observations per condition, this produces N held-out residuals whose root mean square is reported as LOO-RMSE. Because each withheld point is a raw replicate rather than a time-point mean, LOO-RMSE is directly sensitive to replicate-level variability and outlier observations, where an unstable or overfit model will produce large residuals on withheld individual replicates, manifesting as elevated LOO-RMSE. This makes LOO-RMSE the primary criterion for assessing whether model differences are genuine and generalisable rather than artefacts of small-sample instability or in-sample overfitting.

**Estimation of Leave-One-Out Log-Likelihood (LOO-LL):**

Leave-one-out log-likelihood was estimated using a full leave-one-out cross-validation procedure applied at the replicate level. For a dataset containing $n$observations $\left( t_{i} , F_{i} \right)$, each observation $i$was excluded once from the training set. The model was trained on the remaining $n-1$ observations, and a prediction $\hat{F}_{i}^{-i}$ was generated for the held-out time point $t_{i}$. Repeating this procedure for all observations yields a vector of out-of-sample predictions:

$$\hat{F}^{LOO}=\left( \hat{F}_{1}^{-1},\hat{F}_{2}^{-2},\ldots,\hat{F}_{n}^{-n} \right)$$

The residuals between the observed forces and the LOO predictions are then

$$\varepsilon_{i}=F_{i}-\hat{F}_{i}^{-i}$$

Assuming normally distributed residuals, the residual variance is estimated as

$$\sigma^{2}=\frac{1}{n}\sum_{i=1}^{n} \varepsilon_{i}^{2}$$

The leave-one-out log-likelihood is then computed as

$$\mathrm{LOO}\text{-}\mathrm{LL}=-\frac{n}{2}\left[ \log(2\pi\sigma^{2})+1 \right]$$

which corresponds to the Gaussian log-likelihood of the LOO residuals.

Substituting the variance estimate gives

$$\mathrm{LOO}\text{-}\mathrm{LL}=-\frac{n}{2}\left[ \log\left( 2\pi\frac{1}{n}\sum_{i=1}^{n} (F_{i}-\hat{F}_{i}^{-i})^{2} \right)+1 \right]$$

”

**S.5 Neural network architecture, physics regularization, and training configuration of the PINN model**

1. **Neural Network Architecture**

The neural correction term $F_{NN}$ is modeled using a fully connected multilayer perceptron (MLP) with the following structure:

| **Component** | **Specification** |
| --- | --- |
| Input dimension | 1 (normalized time) |
| Hidden layers | 2 |
| Neurons per hidden layer | 32 |
| Activation function | SiLU |
| Output dimension | 1 (correction term $F_{NN}$) |
| Output activation | Linear |
| Network type | Fully connected feed-forward |

Formally, the architecture is:

$$\text{Linear}(1,32)\to\mathrm{SiLU}\to\text{Linear}(32,32)\to\mathrm{SiLU}\to\text{Linear}(32,1)$$

The network output is added to the analytical SLS solution:

$$F_{\text{PINN}}(t)=F_{\text{SLS}}(t)+F_{NN}(t)$$

1. **Physics Regularization**

Physics regularization is enforced through a penalty on:

- The correction magnitude: $\parallel F_{NN}(t)\parallel^{2}$
- Its time derivative: $\parallel\partial F_{NN}/\partial t\parallel^{2}$

The total loss is: $\mathcal{L=}\mathcal{L}_{data}+\mathcal{L}_{physics}$

or, when adaptive weighting is enabled: $\mathcal{L=}e^{-\sigma_{d}}\mathcal{L}_{data}+e^{-\sigma_{p}}\mathcal{L}_{physics}+\sigma_{d}+\sigma_{p}$where $\sigma_{d}$and $\sigma_{p}$are trainable log-variance parameters.

1. **Initialization**

- The MLP weights use PyTorch default initialization (Kaiming uniform for linear layers).
- SLS physical parameters are initialized as:
  - $F_{\max}$: log of maximum observed force
  - $F_{0}$: log of minimum observed force (clipped ≥ 1.0)
  - $\tau$: initialized as log (2)

1. **Optimization and Training**

| **Hyperparameter** | **Value** |
| --- | --- |
| Optimizer | Adam |
| Learning rate | 1e−3 |
| Epochs | 8000 |
| Loss for data term | Smooth L1 (Huber) |
| Explicit weight decay | None |
| Dropout | None |

No additional regularization (e.g., dropout or L2 weight decay) was used. Regularization is provided solely through the physics penalty.

**References**

[1] H. Bettahar, C. Tapeinos, O. Işıtman, C. D’Amico, A. Correia, H.A. Santos, Q. Zhou, Probing Early Particle-Cell Membrane Interactions via Single-Cell and Single-Particle Interaction Analysis, Adv. Funct. Mater. (2025) 2507301. https://doi.org/10.1002/ADFM.202507301;REQUESTEDJOURNAL:JOURNAL:16163028;WGROUP:STRING:PUBLICATION.

[2] S.E. Chesla, P. Selvaraj, C. Zhu, Measuring two-dimensional receptor-ligand binding kinetics by micropipette, Biophys. J. 75 (1998) 1553–1572. https://doi.org/10.1016/S0006-3495(98)74074-3.

[3] J. Zhu, L. Liao, L. Zhu, P. Zhang, K. Guo, J. Kong, C. Ji, B. Liu, Size-dependent cellular uptake efficiency, mechanism, and cytotoxicity of silica nanoparticles toward HeLa cells, Talanta 107 (2013) 408–415. https://doi.org/10.1016/J.TALANTA.2013.01.037.

[4] J. Rejman, V. Oberle, I.S. Zuhorn, D. Hoekstra, Size-dependent internalization of particles via the pathways of clathrin- and caveolae-mediated endocytosis, Biochemical Journal 377 (2004) 159–169. https://doi.org/10.1042/BJ20031253.

[5] J. Han, X.Y. Tong, C.Y. Rao, J.M. Ouyang, B.S. Gui, Size-Dependent Cytotoxicity, Adhesion, and Endocytosis of Micro-/Nano-hydroxyapatite Crystals in HK-2 Cells, ACS Omega 8 (2023) 48432–48443. https://doi.org/10.1021/ACSOMEGA.3C08180/ASSET/IMAGES/LARGE/AO3C08180_0012.JPEG.

[6] L. Ding, C. Yao, X. Yin, C. Li, Y. Huang, M. Wu, B. Wang, X. Guo, Y. Wang, M. Wu, Size, Shape, and Protein Corona Determine Cellular Uptake and Removal Mechanisms of Gold Nanoparticles, Small 14 (2018). https://doi.org/10.1002/SMLL.201801451.

[7] W. Zhang, R. Taheri-Ledari, F. Ganjali, S.S. Mirmohammadi, F.S. Qazi, M. Saeidirad, A. KashtiAray, S. Zarei-Shokat, Y. Tian, A. Maleki, Effects of morphology and size of nanoscale drug carriers on cellular uptake and internalization process: a review, RSC Adv. 13 (2022) 80–114. https://doi.org/10.1039/D2RA06888E.

**Figures**


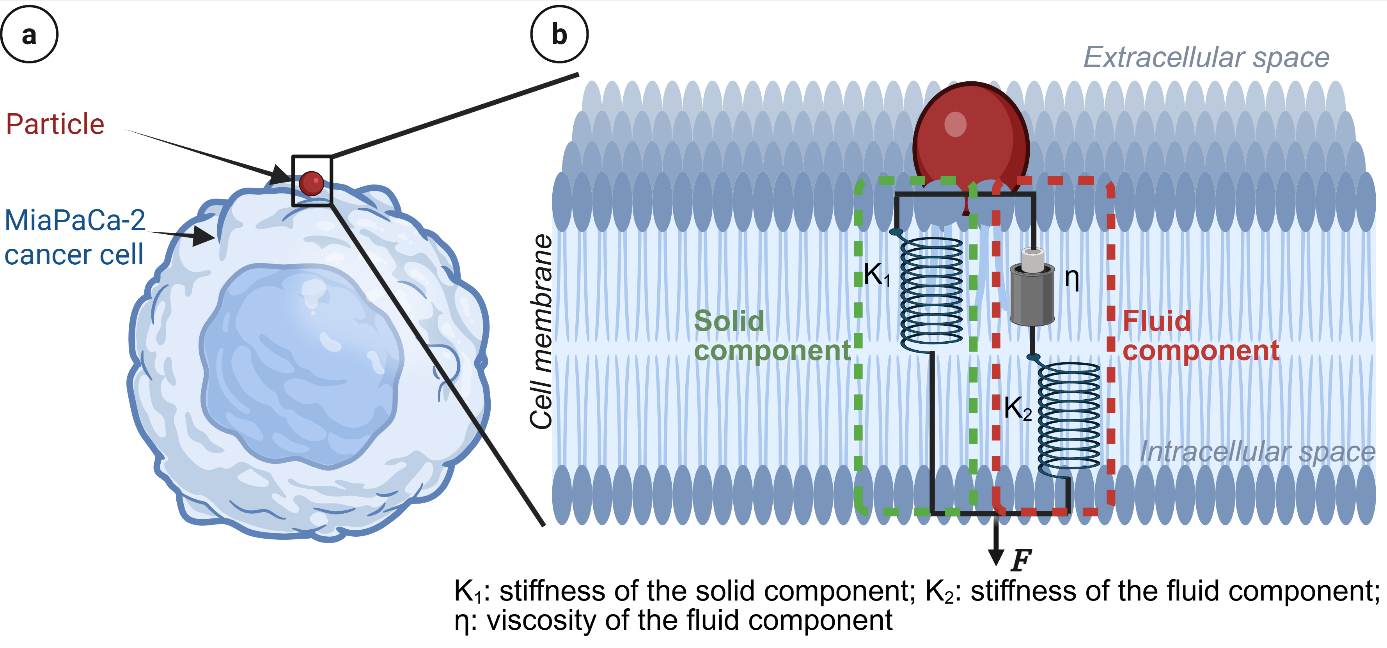


**Figure S1:** Schematic of nanoparticle interaction with a MiaPaCa-2 cancer cell during macropinocytosis. (a) A nanoparticle adheres to the surface of the cell membrane. (b) Zoom-in view illustrating the mechanical response of the membrane modeled as a standard linear solid (SLS) under a cell-generated force$F$, which arises from active internal processes driving particle uptake. The membrane’s behavior is represented by a parallel elastic spring ($k_{1}$​) capturing the solid component, and a Maxwell element (spring $k_{2}$​ in series with dashpot$\eta$) accounting for the viscoelastic fluid component. This model reflects the coupled elastic and time-dependent viscous deformation during cellular internalization of the particle.

**Figure S2:** SLS model training algorithm.

**
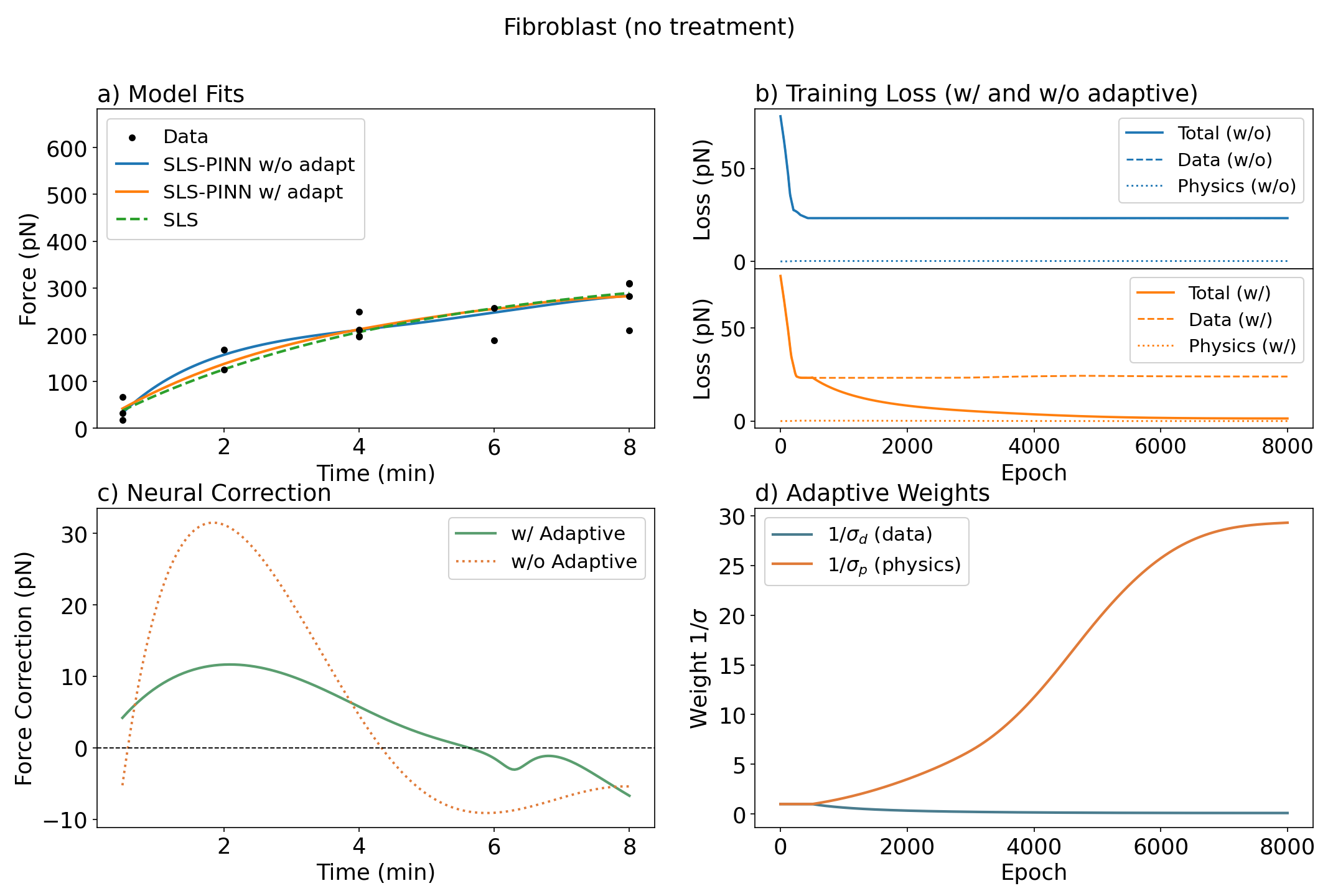
**

**Figure S3:** Model fitting and training dynamics for Fibroblast cells (no treatment). (a) Model fits to traction force measurements over time. Black dots represent experimental data points. The Standard Linear Solid Physics-Informed Neural Network without adaptive weighting (SLS-PINN w/o adapt, blue) and with adaptive weighting (SLS-PINN w/ adapt, orange) are compared against the deterministic SLS baseline (green dashed). (b) Training loss curves for the non-adaptive (top) and adaptive (bottom) PINN variants, decomposed into total loss (solid), data loss (dashed), and physics regularization loss (dotted). (c) Neural correction term, defined as the difference between the PINN prediction and the SLS backbone, for both adaptive and non-adaptive variants. Positive values indicate upward correction relative to the SLS fit. (d) Evolution of the adaptive loss weights $1/\sigma_{d}$(data weight) and $1/\sigma_{p}$(physics weight) over training epochs, reflecting the learned balance between data fidelity and physics regularization.

**
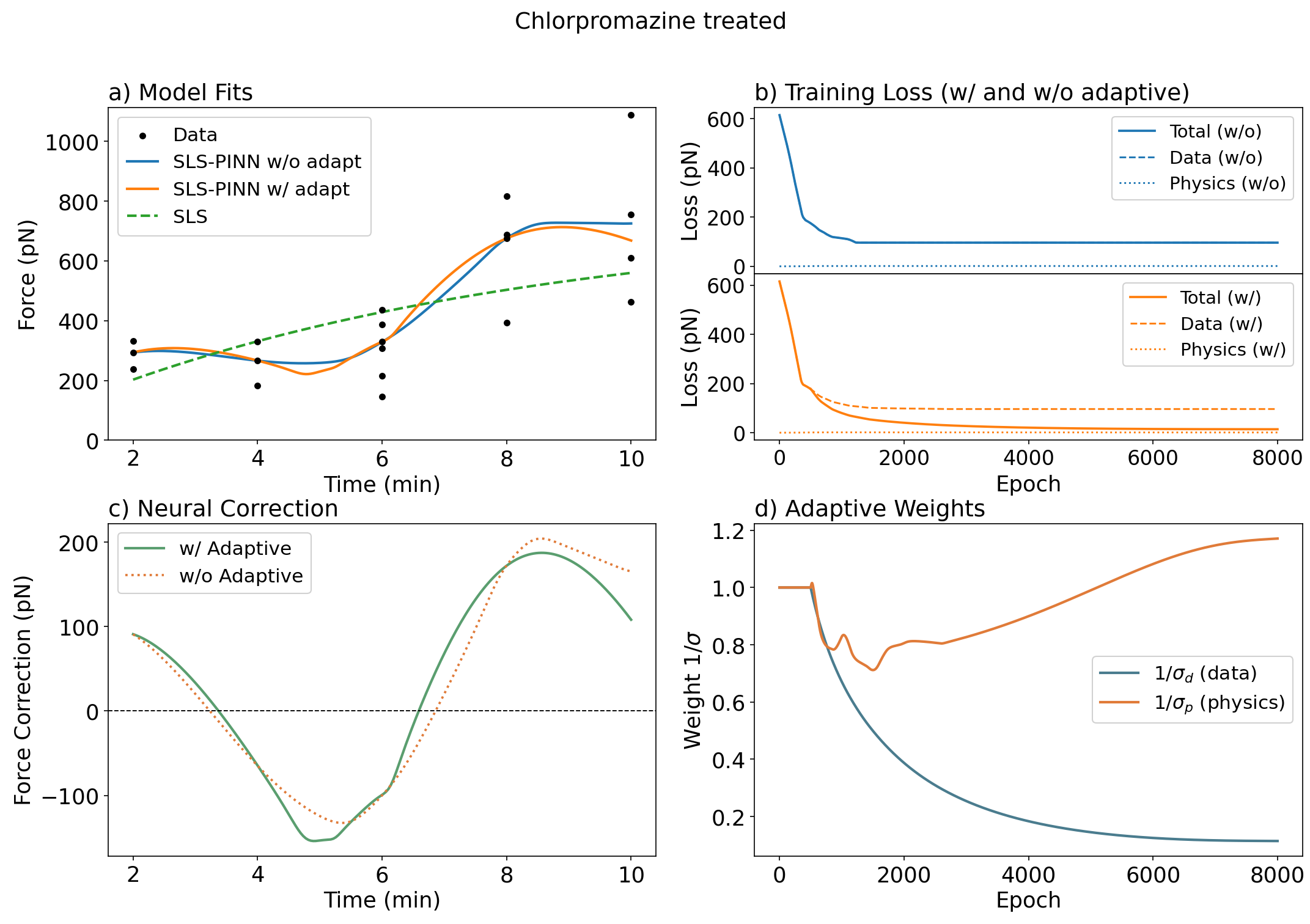
**

**Figure S4**: Model fitting and training dynamics for MiaPaCa-2 cells ( treated with chlorpromazine). (a) Model fits to traction force measurements over time. Black dots represent experimental data points. The Standard Linear Solid Physics-Informed Neural Network without adaptive weighting (SLS-PINN w/o adapt, blue) and with adaptive weighting (SLS-PINN w/ adapt, orange) are compared against the deterministic SLS baseline (green dashed). (b) Training loss curves for the non-adaptive (top) and adaptive (bottom) PINN variants, decomposed into total loss (solid), data loss (dashed), and physics regularization loss (dotted). (c) Neural correction term, defined as the difference between the PINN prediction and the SLS backbone, for both adaptive and non-adaptive variants. Positive values indicate upward correction relative to the SLS fit. (d) Evolution of the adaptive loss weights $1/\sigma_{d}$(data weight) and $1/\sigma_{p}$(physics weight) over training epochs, reflecting the learned balance between data fidelity and physics regularization.

**
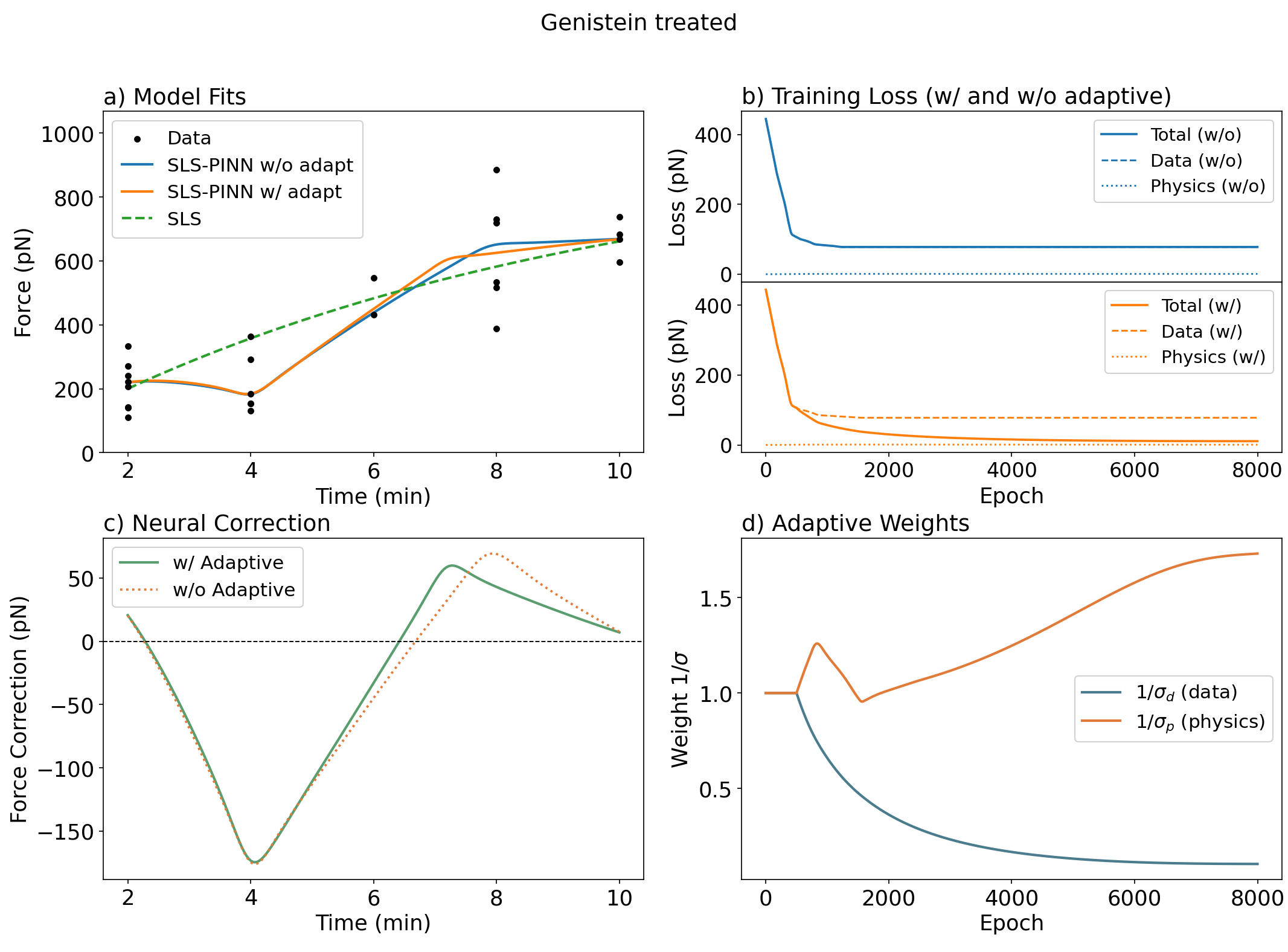
**

**Figure S5:** Model fitting and training dynamics for MiaPaCa-2 cells (treated with Genisten). (a) Model fits to traction force measurements over time. Black dots represent experimental data points. The Standard Linear Solid Physics-Informed Neural Network without adaptive weighting (SLS-PINN w/o adapt, blue) and with adaptive weighting (SLS-PINN w/ adapt, orange) are compared against the deterministic SLS baseline (green dashed). (b) Training loss curves for the non-adaptive (top) and adaptive (bottom) PINN variants, decomposed into total loss (solid), data loss (dashed), and physics regularization loss (dotted). (c) Neural correction term, defined as the difference between the PINN prediction and the SLS backbone, for both adaptive and non-adaptive variants. Positive values indicate upward correction relative to the SLS fit. (d) Evolution of the adaptive loss weights $1/\sigma_{d}$(data weight) and $1/\sigma_{p}$(physics weight) over training epochs, reflecting the learned balance between data fidelity and physics regularization.

**
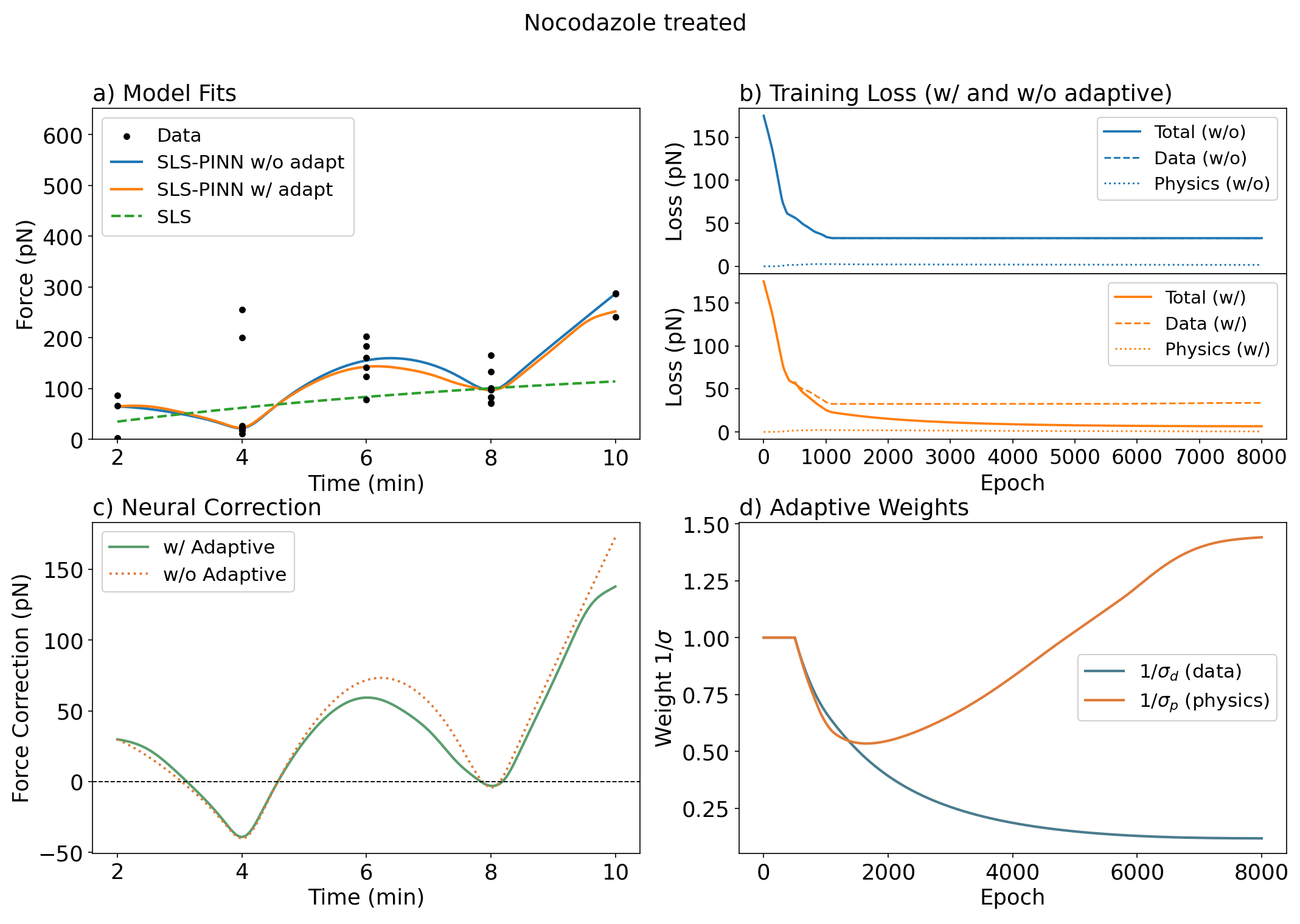
**

**Figure S6:** Model fitting and training dynamics for MiaPaCa-2 cells (treated with Nocodazole). (a) Model fits to traction force measurements over time. Black dots represent experimental data points. The Standard Linear Solid Physics-Informed Neural Network without adaptive weighting (SLS-PINN w/o adapt, blue) and with adaptive weighting (SLS-PINN w/ adapt, orange) are compared against the deterministic SLS baseline (green dashed). (b) Training loss curves for the non-adaptive (top) and adaptive (bottom) PINN variants, decomposed into total loss (solid), data loss (dashed), and physics regularization loss (dotted). (c) Neural correction term, defined as the difference between the PINN prediction and the SLS backbone, for both adaptive and non-adaptive variants. Positive values indicate upward correction relative to the SLS fit. (d) Evolution of the adaptive loss weights $1/\sigma_{d}$(data weight) and $1/\sigma_{p}$(physics weight) over training epochs, reflecting the learned balance between data fidelity and physics regularization.


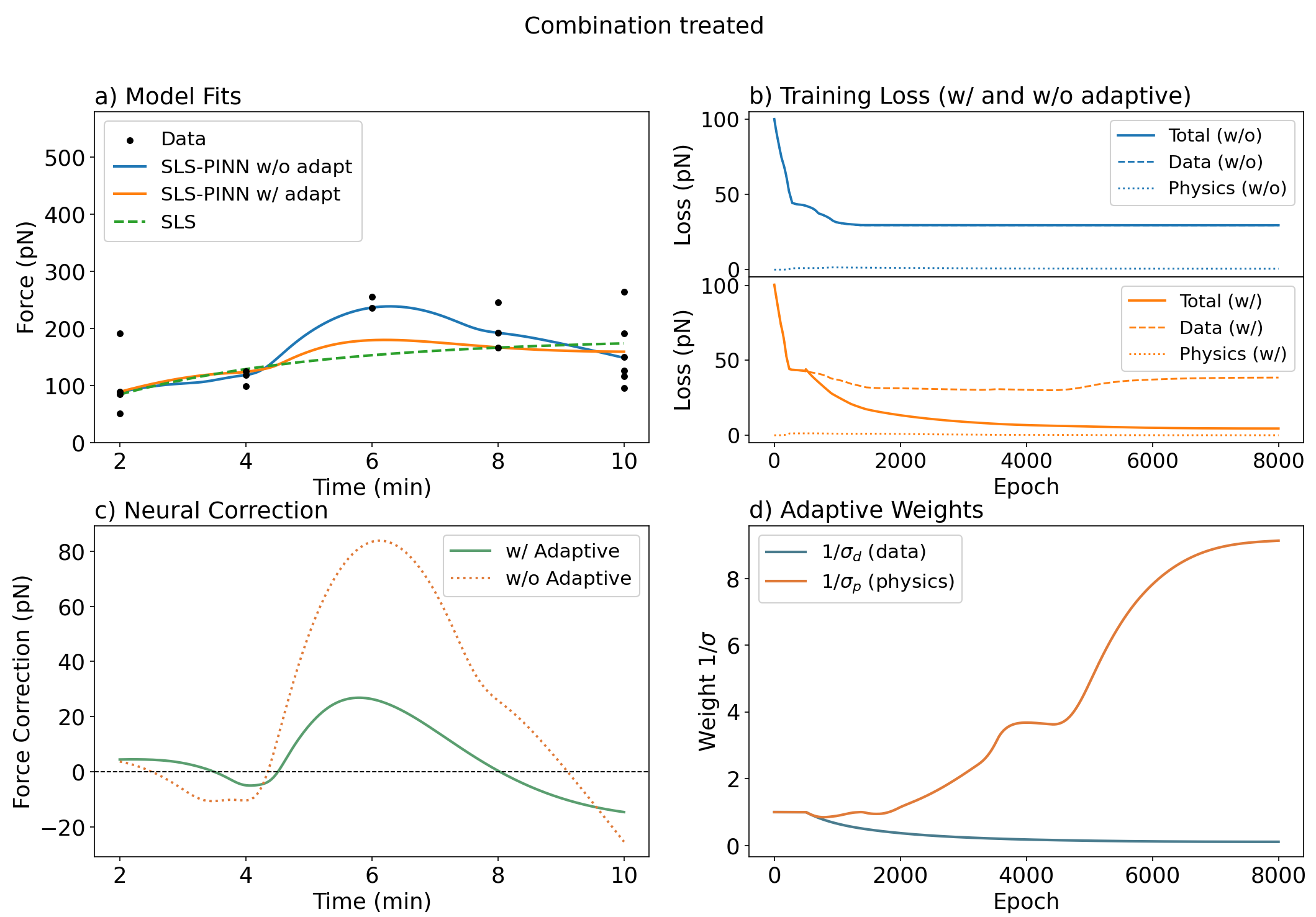


**Figure S7:** Model fitting and training dynamics for MiaPaCa-2 cells (treated with the combination of the three inhibitors: chlorpromazine, genistein, nocodazole). (a) Model fits to traction force measurements over time. Black dots represent experimental data points. The Standard Linear Solid Physics-Informed Neural Network without adaptive weighting (SLS-PINN w/o adapt, blue) and with adaptive weighting (SLS-PINN w/ adapt, orange) are compared against the deterministic SLS baseline (green dashed). (b) Training loss curves for the non-adaptive (top) and adaptive (bottom) PINN variants, decomposed into total loss (solid), data loss (dashed), and physics regularization loss (dotted). (c) Neural correction term, defined as the difference between the PINN prediction and the SLS backbone, for both adaptive and non-adaptive variants. Positive values indicate upward correction relative to the SLS fit. (d) Evolution of the adaptive loss weights $1/\sigma_{d}$(data weight) and $1/\sigma_{p}$(physics weight) over training epochs, reflecting the learned balance between data fidelity and physics regularization.


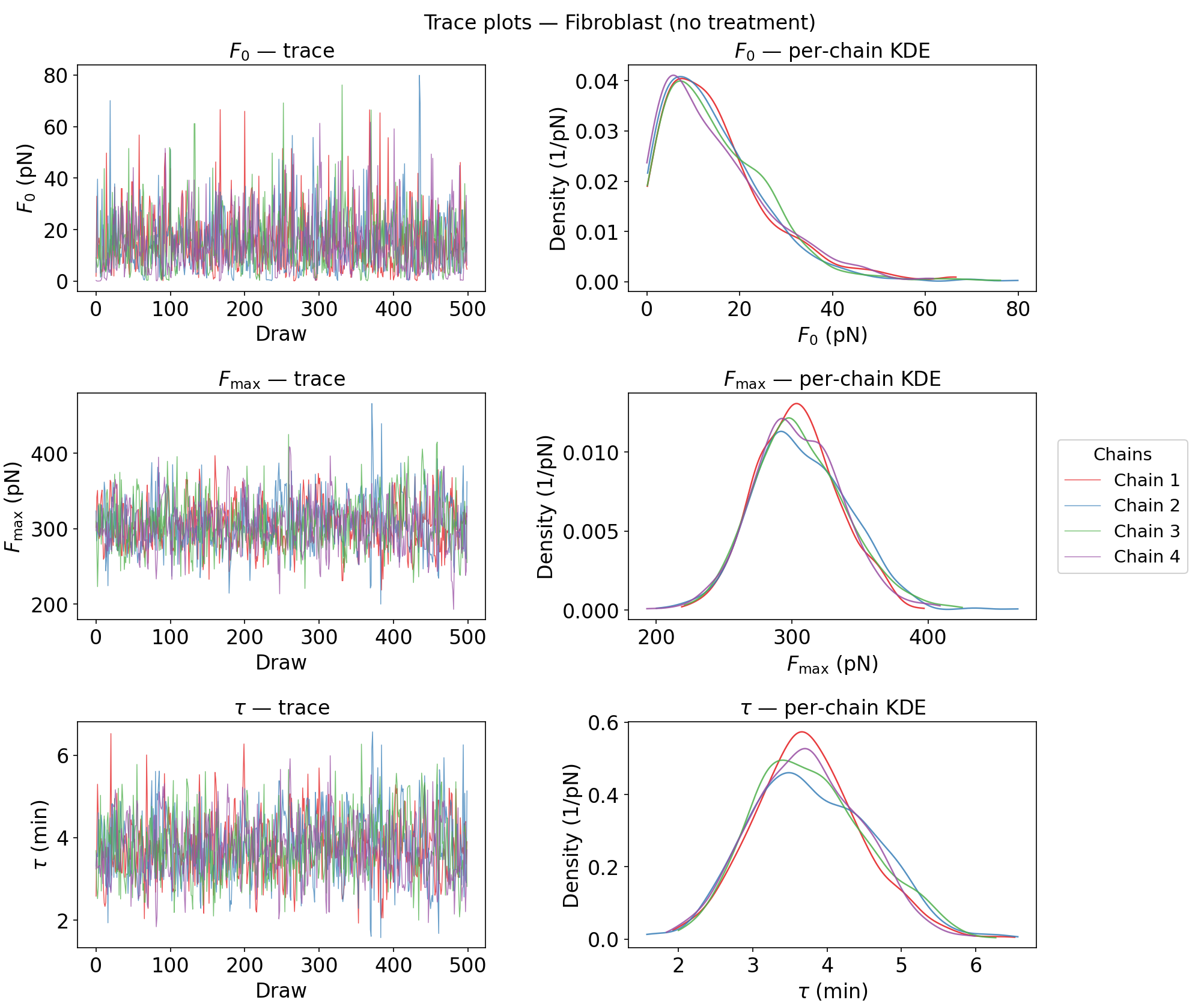


**Figure S8:** MCMC trace plots and per-chain posterior distributions for untreated fibroblast cells. Each row corresponds to one kinetic parameter: initial effective contact force $F₀$ (top), effective maximum adhesion force $F_{max}$ (middle), and effective characteristic adhesion time constant $\tau$ (bottom). Left column: trace plots showing sampled parameter values across 500 draws for each of the 4 independent MCMC chains; stationary traces with no visible trends or drifts confirm that all chains reached the target distribution. Right column: marginal posterior kernel density estimates (KDE) per chain; the close overlap of the four KDE curves confirms convergence to a common posterior distribution. Posterior sampling was performed using the No-U-Turn Sampler (NUTS) with 4 independent chains, 500 warmup steps, and 500 draws per chain (2000 total posterior samples). Gelman-Rubin $\hat{R}$< 1.01 and bulk effective sample size ESS > 400 were confirmed for all three parameters.


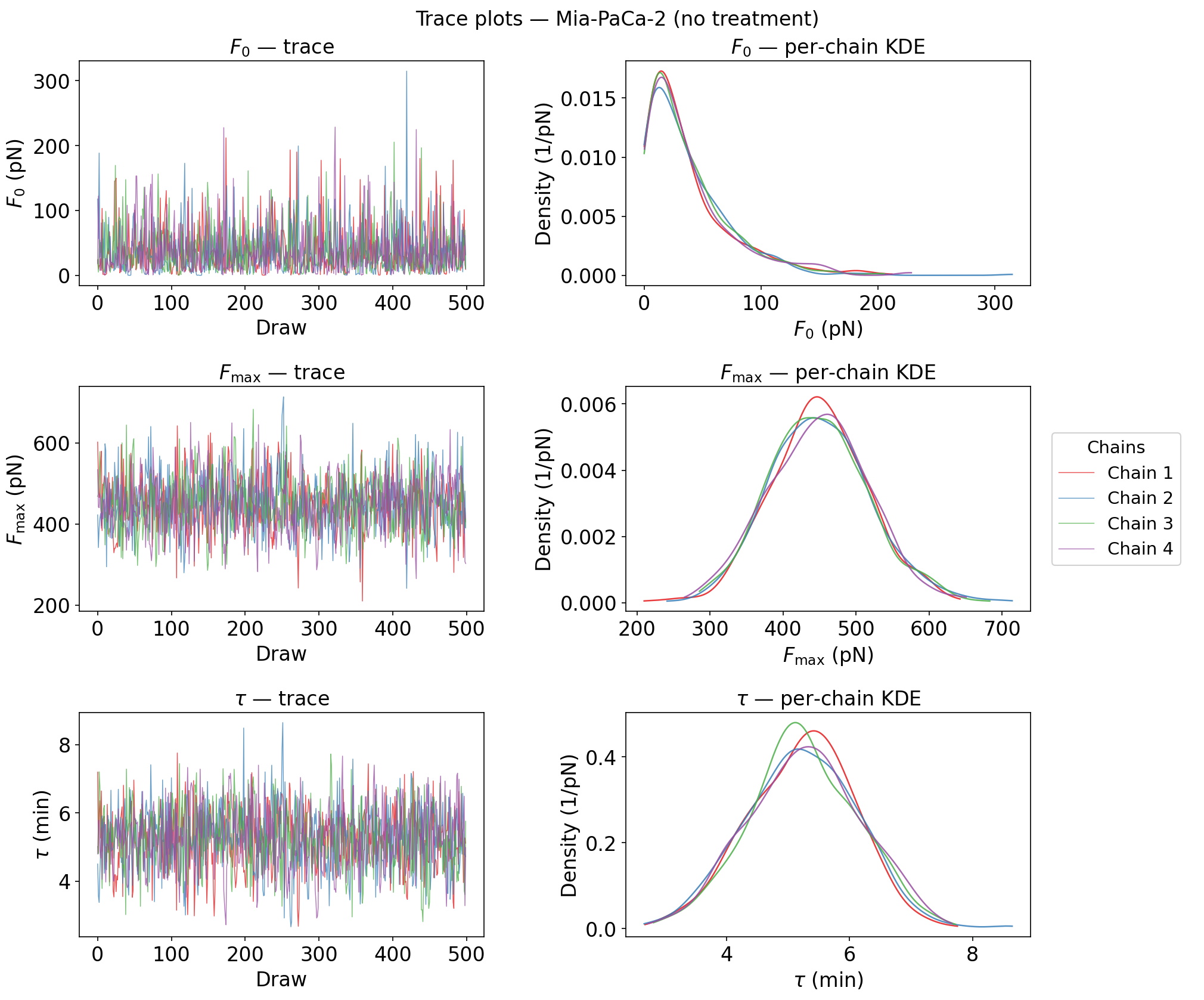


**Figure S9:** MCMC trace plots and per-chain posterior distributions for untreated Mia-PaCa-2 cells. Each row corresponds to one kinetic parameter: initial effective contact force $F₀$ (top), effective maximum adhesion force $F_{max}$ (middle), and effective characteristic adhesion time constant $\tau$ (bottom). Left column: trace plots showing sampled parameter values across 500 draws for each of the 4 independent MCMC chains; stationary traces with no visible trends or drifts confirm that all chains reached the target distribution. Right column: marginal posterior kernel density estimates (KDE) per chain; the close overlap of the four KDE curves confirms convergence to a common posterior distribution. Posterior sampling was performed using the No-U-Turn Sampler (NUTS) with 4 independent chains, 500 warmup steps, and 500 draws per chain (2000 total posterior samples). Gelman-Rubin $\hat{R}$ < 1.01 and bulk effective sample size ESS > 400 were confirmed for all three parameters.


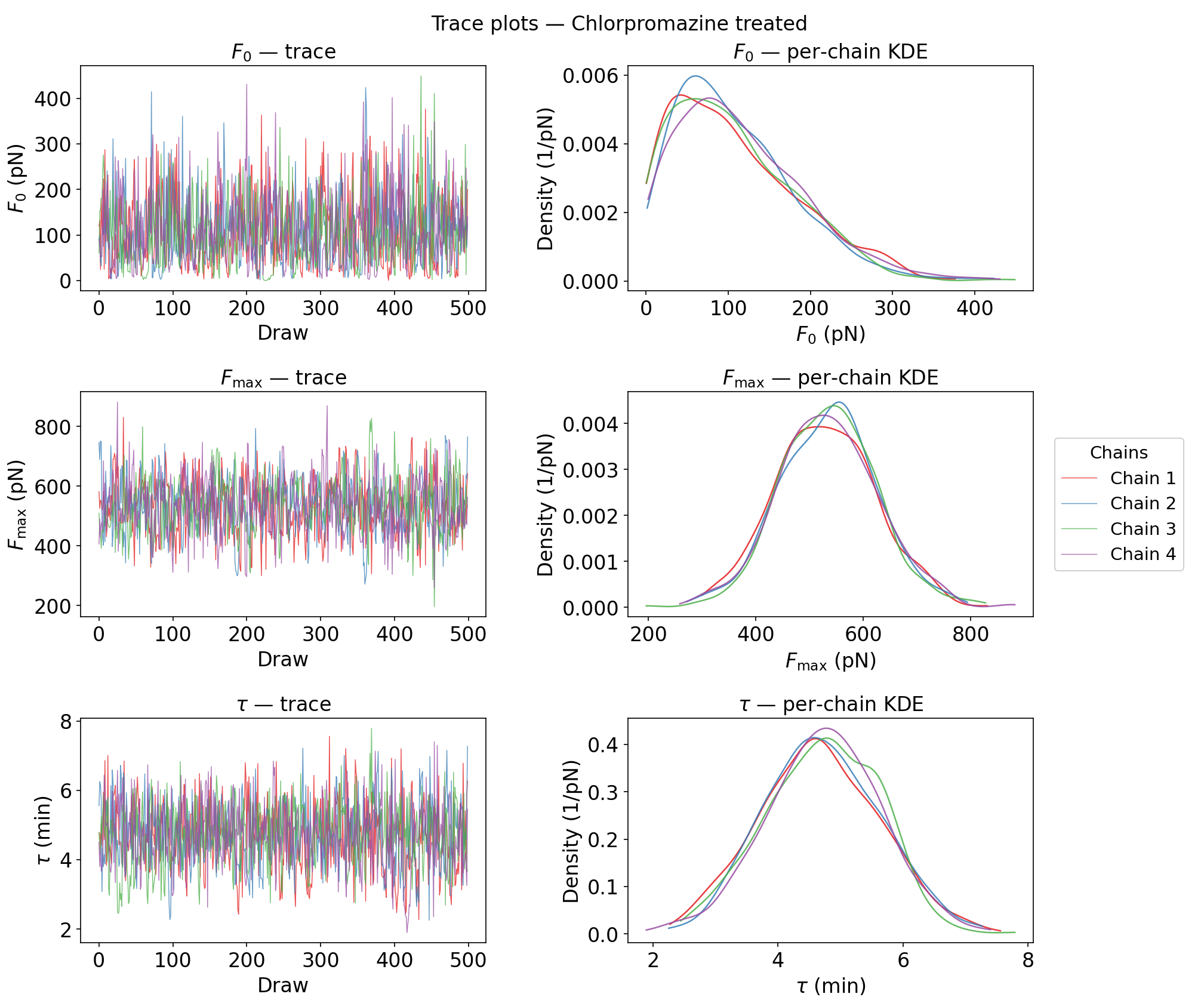


**Figure S10:** MCMC trace plots and per-chain posterior distributions for Mia-PaCa-2 cells treated with Chlorpromazine. Each row corresponds to one kinetic parameter: initial effective contact force $F₀$ (top), effective maximum adhesion force $F_{max}$ (middle), and effective characteristic adhesion time constant $\tau$ (bottom). Left column: trace plots showing sampled parameter values across 500 draws for each of the 4 independent MCMC chains; stationary traces with no visible trends or drifts confirm that all chains reached the target distribution. Right column: marginal posterior kernel density estimates (KDE) per chain; the close overlap of the four KDE curves confirms convergence to a common posterior distribution. Posterior sampling was performed using the No-U-Turn Sampler (NUTS) with 4 independent chains, 500 warmup steps, and 500 draws per chain (2000 total posterior samples). Gelman-Rubin $\hat{R}$< 1.01 and bulk effective sample size ESS > 400 were confirmed for all three parameters.


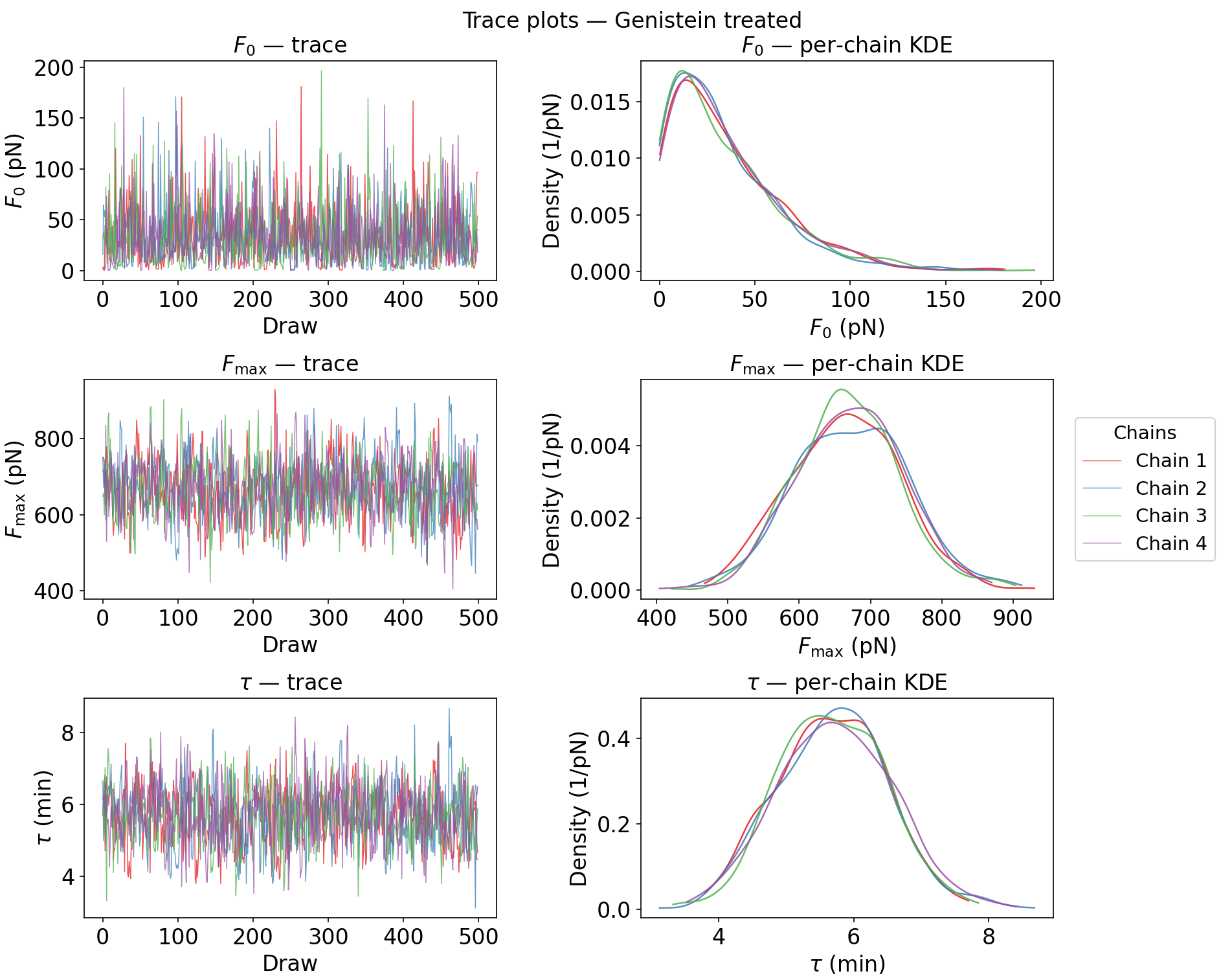


**Figure S 11:** MCMC trace plots and per-chain posterior distributions for Mia-PaCa-2 cells treated with Genistein. Each row corresponds to one kinetic parameter: initial effective contact force $F₀$ (top), effective maximum adhesion force $F_{max}$ (middle), and effective characteristic adhesion time constant $\tau$ (bottom). Left column: trace plots showing sampled parameter values across 500 draws for each of the 4 independent MCMC chains; stationary traces with no visible trends or drifts confirm that all chains reached the target distribution. Right column: marginal posterior kernel density estimates (KDE) per chain; the close overlap of the four KDE curves confirms convergence to a common posterior distribution. Posterior sampling was performed using the No-U-Turn Sampler (NUTS) with 4 independent chains, 500 warmup steps, and 500 draws per chain (2000 total posterior samples). Gelman-Rubin R$\hat{R}$ < 1.01 and bulk effective sample size ESS > 400 were confirmed for all three parameters.


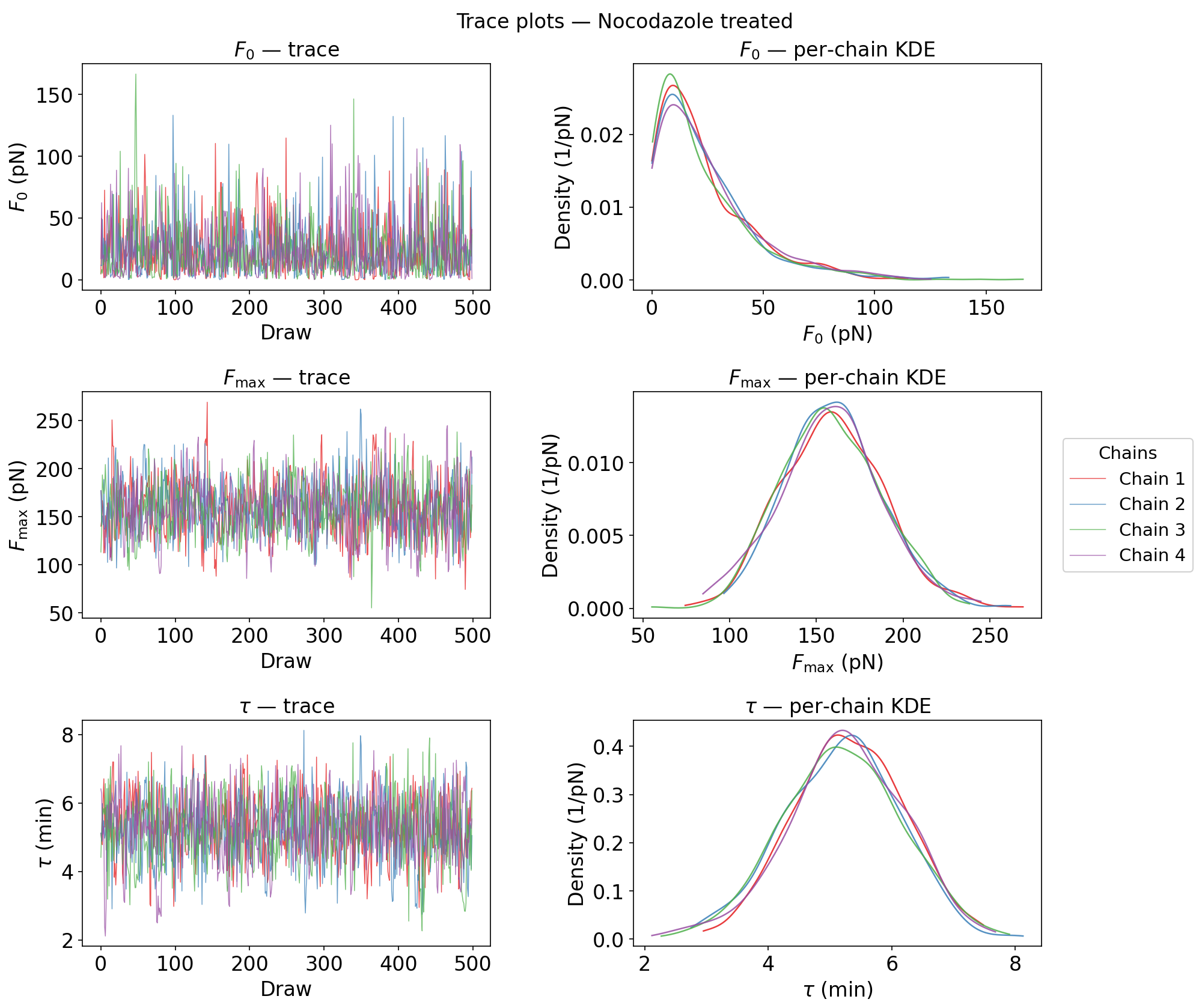


**Figure S12:** MCMC trace plots and per-chain posterior distributions for Mia-PaCa-2 cells treated with Nocodazole**.** Each row corresponds to one kinetic parameter: initial effective contact force $F₀$ (top), effective maximum adhesion force $F_{max}$ (middle), and effective characteristic adhesion time constant $\tau$ (bottom). Left column: trace plots showing sampled parameter values across 500 draws for each of the 4 independent MCMC chains; stationary traces with no visible trends or drifts confirm that all chains reached the target distribution. Right column: marginal posterior kernel density estimates (KDE) per chain; the close overlap of the four KDE curves confirms convergence to a common posterior distribution. Posterior sampling was performed using the No-U-Turn Sampler (NUTS) with 4 independent chains, 500 warmup steps, and 500 draws per chain (2000 total posterior samples). Gelman-Rubin $\hat{R}$ < 1.01 and bulk effective sample size ESS > 400 were confirmed for all three parameters.


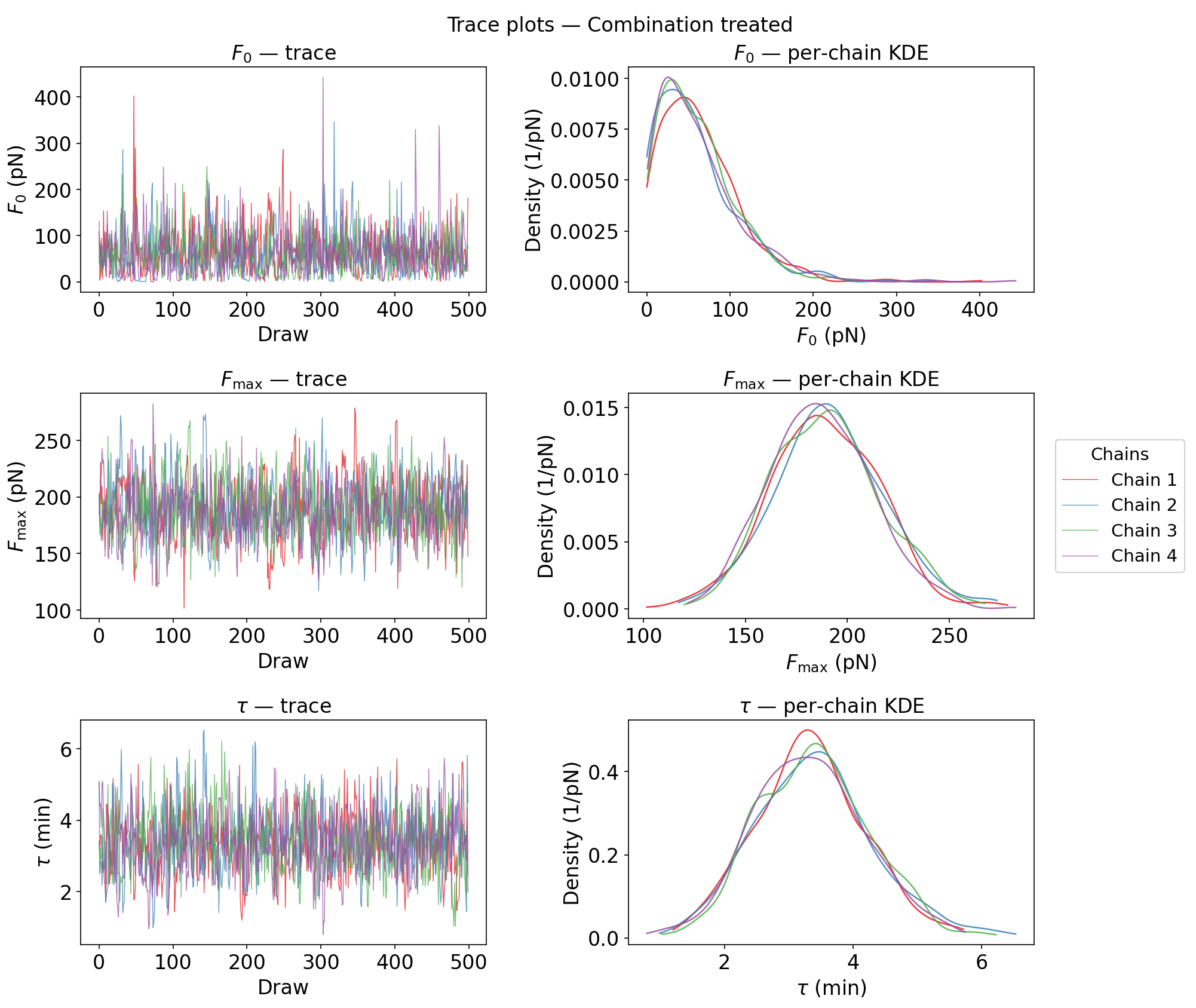


**Figure S13:** MCMC trace plots and per-chain posterior distributions for Mia-PaCa-2 cells treated with a combination of all three inhibitors (3 Inhibitors). Each row corresponds to one kinetic parameter: initial effective contact force $F₀$ (top), effective maximum adhesion force $F_{max}$ (middle), and effective characteristic adhesion time constant $\tau$ (bottom). Left column: trace plots showing sampled parameter values across 500 draws for each of the 4 independent MCMC chains; stationary traces with no visible trends or drifts confirm that all chains reached the target distribution. Right column: marginal posterior kernel density estimates (KDE) per chain; the close overlap of the four KDE curves confirms convergence to a common posterior distribution. Posterior sampling was performed using the No-U-Turn Sampler (NUTS) with 4 independent chains, 500 warmup steps, and 500 draws per chain (2000 total posterior samples). Gelman-Rubin $\hat{R}$ < 1.01 and bulk effective sample size ESS > 400 were confirmed for all three parameters.


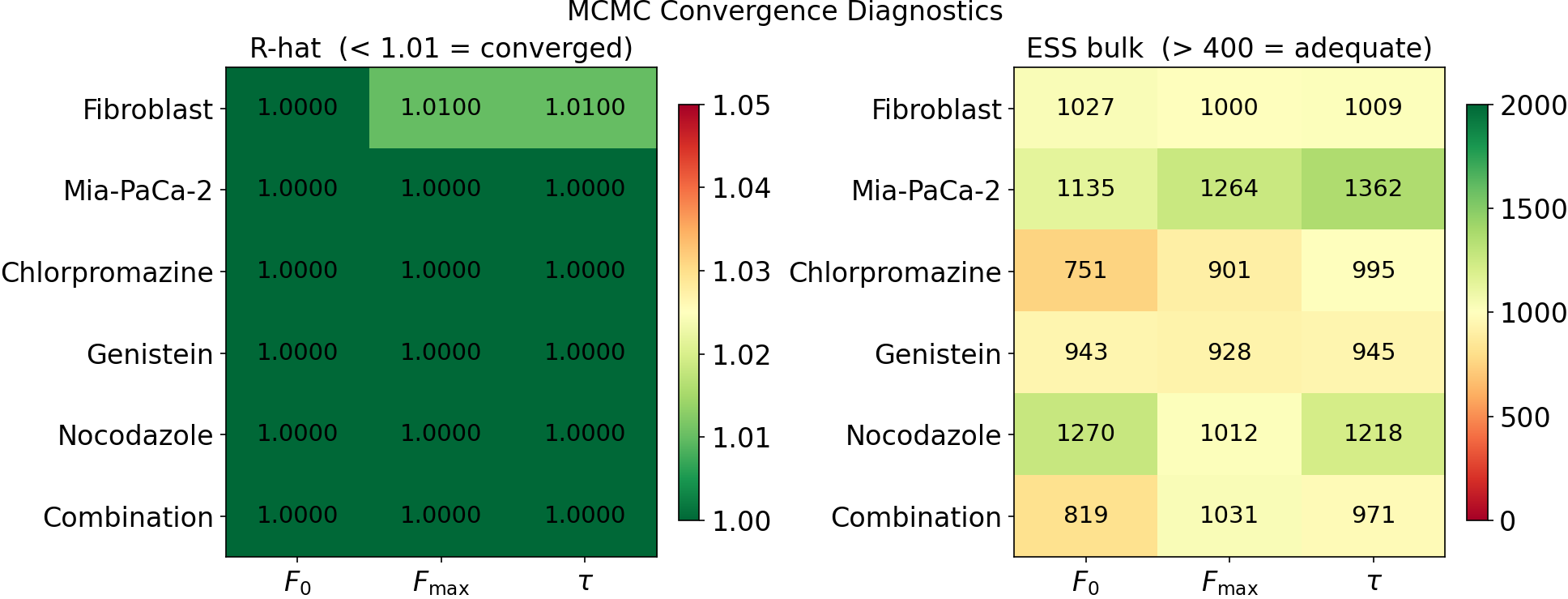


**Figure S14**: **MCMC convergence diagnostics for all experimental conditions and kinetic parameters.** Left panel: Gelman-Rubin R-hat statistics for the three kinetic parameters ( initial effective contact force $F₀$, effective maximum adhesion force $F_{m\mathrm{ax}}$, and effective characteristic adhesion time constant $\tau$ ) across all six experimental conditions. All values are ≤ 1.01, confirming adequate convergence of all four independent MCMC chains to a common posterior distribution. Right panel: bulk effective sample size (ESS_bulk) for the same parameters and conditions. All ESS bulk values exceed 600, substantially above the recommended threshold of 400, indicating adequate posterior exploration and reliable estimation of posterior means and credible intervals. The lowest ESS bulk observed is 751 for $F₀$under Chlorpromazine treatment, consistent with the higher replicate variability and broader posterior uncertainty in this condition. Posterior sampling was performed using the No-U-Turn Sampler (NUTS) with 4 independent chains, 500 warmup steps, and 500 draws per chain (2000 total posterior samples per condition).


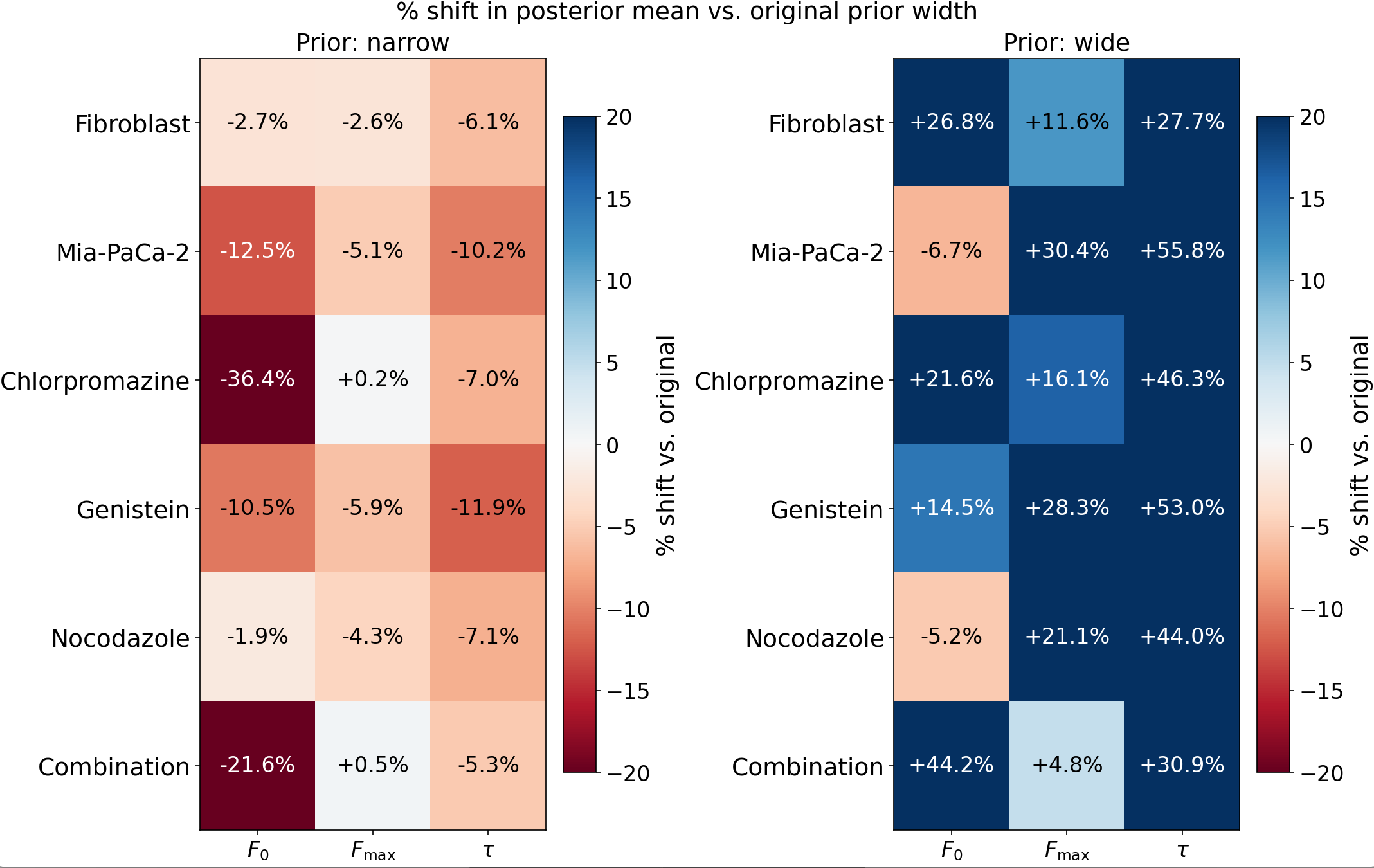


**Figure S15:** Percentage shift in posterior mean relative to the original prior width ($\sigma_{F_{max}^{*}}$ , $\sigma_{F_{0}^{*}}$ = 300 pN , $\sigma_{\tau^{*}}$=1 min) under narrow (left, $\sigma_{F_{max}^{*}}$ , $\sigma_{F_{0}^{*}}$ = 50 pN, $\sigma_{\tau^{*}}$=0.5 min) and wide (right, $\sigma_{F_{max}^{*}}$ , $\sigma_{F_{0}^{*}}$ = 600 pN , $\sigma_{\tau^{*}}$=3 min). ) prior-width regimes, across all six experimental conditions and three kinetic parameters ($F_{0}$, $F_{m\mathrm{ax}}$, τ). Values near zero (white) confirm posterior insensitivity to prior specification. Large positive values for τ under the wide prior (up to +56%) in data-sparse conditions reflect the finite observation window (t ≤ 10 min), where the exponential saturation is not fully resolved and the likelihood cannot strongly constrain τ.


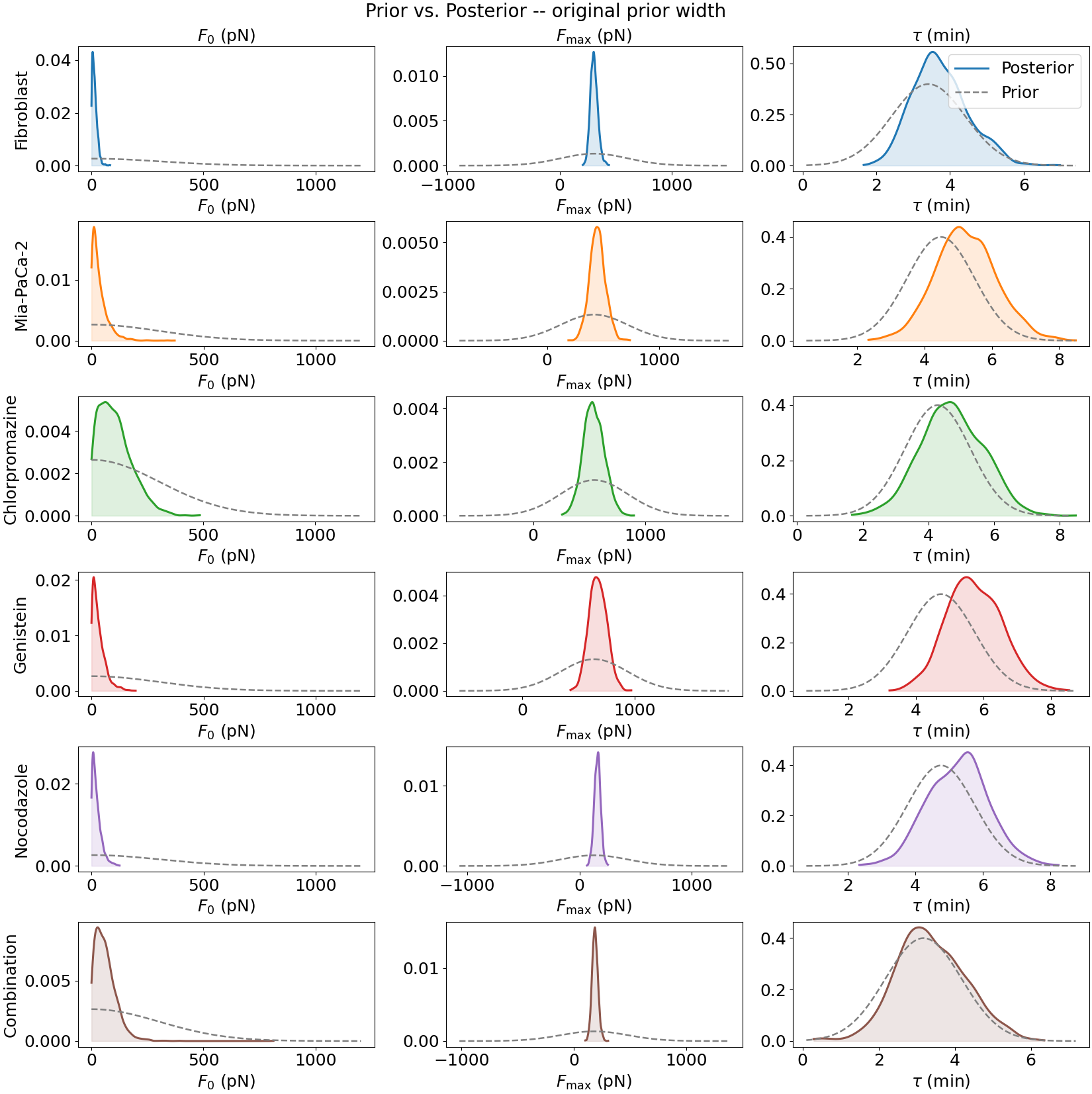


**Figure S16:** Prior (dashed grey) versus posterior (solid coloured) distributions for $F_{0}$(left), $F_{m\mathrm{ax}}$(centre), and $\tau$ (right) under the original prior width ($\sigma_{F_{max}^{*}}$ , $\sigma_{F_{0}^{*}}$ = 300 pN , $\sigma_{\tau^{*}}$=1 min) across all six experimental conditions. For $F_{0}$and $F_{m\mathrm{ax}}$, the posterior is dramatically narrower than the prior in all conditions, confirming likelihood-dominated inference. For τ, the posterior is moderately narrower than the prior, consistent with partial identifiability near the observation window boundary. Distributions were estimated from 2000 pooled posterior samples (4 chains × 500 draws) after confirming convergence via $\hat{R}$< 1.01 and ESS > 400.


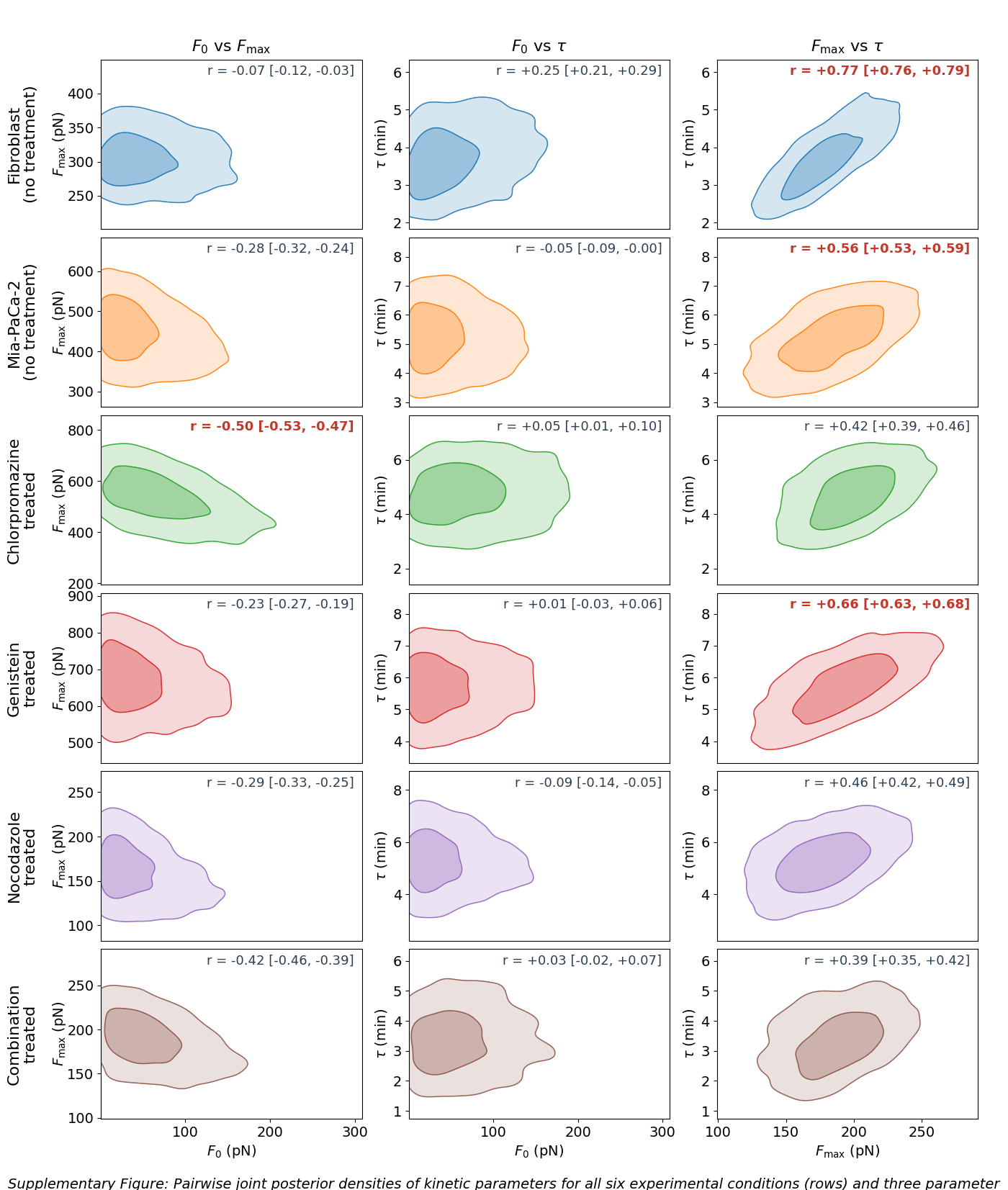


**Figure S17:** Pairwise joint posterior densities of kinetic parameters for all six experimental conditions (rows) and three parameter pairs (columns). Filled contours mark the 50 % (darker) and 90 % (lighter) posterior probability-mass regions estimated by 2-D kernel density estimation. Pearson correlation coefficient r with its 95 % confidence interval is annotated in the upper corner of each panel (bold red: |r| > 0.5; dark grey: |r| ≤ 0.5). Sampling: 4 chains × 500 draws (2000 total posterior samples per condition).
